# A New Paper-Based Microfluidic Device for Improved Detection of Nitrate in Water

**DOI:** 10.3390/s21010102

**Published:** 2020-12-26

**Authors:** Amer Charbaji, Hojat Heidari-Bafroui, Constantine Anagnostopoulos, Mohammad Faghri

**Affiliations:** Microfluidics Laboratory, Department of Mechanical, Industrial and Systems Engineering, University of Rhode Island, 2 East Alumni Avenue, Kingston, RI 02881, USA; h_heidari@uri.edu (H.H.-B.); anagnostopoulos@uri.edu (C.A.)

**Keywords:** paper-based devices, paper microfluidics, colorimetric assay, nitrate detection, nitrite detection, Griess reaction, zinc microparticles, point-of-care diagnostics, sensors, low-cost platforms

## Abstract

In this paper, we report a simple and inexpensive paper-based microfluidic device for detecting nitrate in water. This device incorporates two recent developments in paper-based technology suitable for nitrate detection and has an optimized microfluidic design. The first technical advancement employed is an innovative fibrous composite material made up of cotton fibers and zinc microparticles that can be incorporated in paper-based devices and results in better nitrate reduction. The second is a detection zone with an immobilized reagent that allows the passage of a larger sample volume. Different acids were tested—citric and phosphoric acids gave better results than hydrochloric acid since this acid evaporates completely without leaving any residue behind on paper. Different microfluidic designs that utilize various fluid control technologies were investigated and a design with a folding detection zone was chosen and optimized to improve the uniformity of the signal produced. The optimized design allowed the device to achieve a limit of detection and quantification of 0.53 ppm and 1.18 ppm, respectively, for nitrate in water. This accounted for more than a 40% improvement on what has been previously realized for the detection of nitrate in water using paper-based technology.

## 1. Introduction

Paper-based microfluidic technology has seen a great deal of advancements over the past several years due to the growing interest in the many advantages they provide, most notably, their low cost, portability, deployability, ease of use, and disposability. Most recent advancements in paper-based technology were for water analysis [1,2,3], biomedical applications [4,5,6], food safety analysis [7,8], soil analysis [9], and in many other applications [10]. Although paper has been used for biological and chemical applications for over a century with the simple use of litmus paper, paper chromatography, and dried blood cells [11,12], only recently have more complex systems (lab-on-paper) been developed and achieved on paper-based devices. The first simple paper-based microfluidic device may be attributed to that mentioned by Muller et al. in 1949 [13,14,15], but it was the Whitesides group [16] who showed the promise and endless possibilities of this technology. Paper-based devices make use of capillary action to flow fluids through paper without the use of a pump. This removes the need for an external power source to drive the fluid and results in product miniaturization and major cost savings. 

Paper-based microfluidic devices are made up of multiple sections that serve different purposes. The simpler devices generally have a sample port, onto which the sample fluid is loaded; transport channels, which connect the different sections of the device; reaction zones, at which the sample fluid reacts or mixes with dry or wet reagents; and a detection zone, at which a signal is formed that can be either qualitative in nature or can be measured quantitatively. The majority of paper-based devices utilize colorimetric reactions to produce a quantifiable signal [17]. Plenty of papers have already been published on these devices, including various fabrication methods and sensing techniques [18,19,20].

While a large number of applications have been described and implemented using paper-based microfluidic devices, opportunities for improving their performance still exist due to ongoing advancements in the field of paper-based technology. An example is the performance of a paper-based microfluidic device for the detection of nitrate. Nitrate is a naturally occurring ion that is part of the nitrogen cycle [21]. It is also an essential nutrient needed for plant growth but plays a significant role in water nutrient pollution when present at elevated concentrations [22]. Nitrate in water emanates from several different sources with large quantities coming from fertilizer or manure runoff, atmospheric deposition, agricultural sources, septic tanks, and wastewater treatment plants. Since nitrate is the most stable form of nitrogen in oxygenated environments, all other forms of nitrogen-containing compounds in water can also become sources for dissolved nitrate [23,24]. Drinking water with high levels of nitrate increases the risk of developing colorectal cancer, thyroid disease, and central nervous system birth defects [25]. The United States Environmental Protection Agency (U.S. EPA) has set the maximum contaminant level of nitrate in drinking water as 10 ppm; however, concentrations greater than 3 ppm indicate contamination of the groundwater and those greater than 1 ppm indicate human activity [26]. As monitoring the quality of surface, ground, and drinking water has become a major concern in present times [27], measuring nitrate levels in water for environmental protection purposes and to ensure its safety and suitability for consumption has become even more pressing. There are several detection techniques currently in use to measure nitrate levels in water [28]; however, these conventional methods require costly instruments and time consuming analysis [29]. They also require special sample handling and preparation, which requires trained personnel [30]. Therefore, microfluidic technology for water quality analysis, which includes paper-based technology, has been growing as it provides several advantages including rapid and economical detection techniques [31,32]. Thus far, only five paper-based microfluidic devices have been developed for the detection of nitrate. These devices use the Griess assay for the colorimetric detection of nitrate in water [33], food samples [34,35,36], and human saliva [37]. Since the Griess assay is specific to nitrite, nitrate molecules have to be first reduced to nitrite before undergoing the reaction. The majority of these devices used zinc microparticles to achieve this reduction. However, there have been two recent developments in paper-based microfluidic technology. The first is a new composite material, which we developed in [38], which increases the reduction efficiency of nitrate to nitrite. The second is a functionalized paper, one that has an immobilized Griess reagent [39], which allows the passage of a larger volume of sample over the detection zone. Moreover, device architecture plays a major role in paper-based microfluidics. Primarily, proper control of fluid flow through the different sections of the device is of great importance to achieve the required reduction or reaction times. The design chosen for the device impacts the quality and uniformity of the signal developed in the detection zone. This largely affects the performance of the device and the limits of detection attained.

In this work, we follow an engineering approach by incorporating these two latest innovations in a new paper-based microfluidic device to improve nitrate detection in water samples. The final optimized architecture employs a folding design that allows for a more uniform color to develop in the detection zone. Results show an enhancement of over 40% in the detection and quantification limits of nitrate in water compared to what has been previously achieved using paper-based technology. It is worth mentioning that different paper-based microfluidic designs were initially developed and tested before and after employing the two innovations. These designs are briefly discussed in the Supplementary Information document, which gives some of the advantages and disadvantages observed for each design. This should aid other researchers when developing paper-based devices for any application of interest.

## 2. Materials and Methods

### 2.1. The Greiss Assay 

The Griess reaction was first described in 1864 and demonstrated suitable for the detection of nitrite in 1879 [40]. It is the most commonly used spectrophotometric method for quantifying concentrations of nitrate and nitrite [41,42]. The Griess assay involves 2 reaction steps that take place under acidic conditions. In this reaction, nitrite molecules have to first react with sulfanilamide to form diazonium ions (Equation (1) below). These ions then react with naphthyl ethylene diamine (NED) molecules to produce a visible azo dye, which is pinkish red in color (Equation (2)). Therefore, nitrate has to be reduced to nitrite first before being detected by the Griess assay. There are different reducing agents that can be used to reduce nitrate to nitrite. The most commonly used reducing agents are cadmium, copperized cadmium, zinc, nitrate reductase, hydrazine sulfate, titanium (III) chloride, or vanadium (III) [28,31]. Cadmium reduction is the leading method used for nitrate detection; however, more researchers are using zinc since it is a less toxic reductant and not as harmful to humans or the environment as cadmium [43,44]. Moreover, it is worth mentioning that Jayawardane et al. [33] obtained similar results for nitrate detection when using cadmium or zinc in a paper-based microfluidic device. Therefore, we used zinc to reduce nitrate to nitrite (Equation (3)).

(1) $$\mathrm{Sulfanilamide}\;+{\;\mathrm{NO}}_{2}^{-}\overset{acid}{\to }\;\mathrm{Diazonium}\;\mathrm{salt}$$

(2) $$\mathrm{Diazonium}\;\mathrm{salt}\;+\;N-\left(1\mathrm{naphthyl}\right)\mathrm{ethylenediamine}\;\overset{acid}{\to }\;\mathrm{Pinkish}-\mathrm{red}\;\mathrm{azo}\;\mathrm{dye}$$

(3) $${\mathrm{NO}}_{3}^{-}\;+\;2H^{+}+\;2e^{-}\;\overset{Zinc}{\to }{\;\mathrm{NO}}_{2}^{-}+{\;H}_{2}O$$

### 2.2. A New Composite Material That Improves Nitrate Conversion Efficiency 

**Figure 1:**
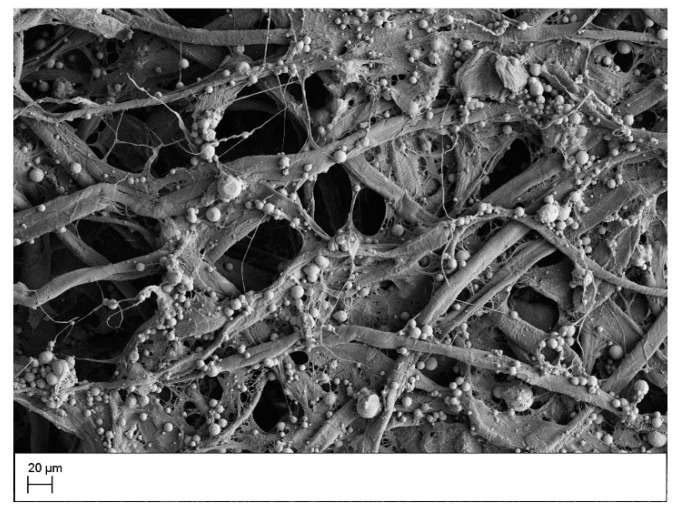
A scanning electron microscope image of a Zinculose strip at 500×.

Zinculose, a recently developed composite material, is made up of cotton fibers with zinc microparticles embedded within the matrix of the material. It allows for a greater contact area between nitrate molecules and the zinc microparticles since these particles are directly present along the flow path of the sample and not simply sitting on the surface of the paper (Figure 1). Zinculose can be produced by a simple and inexpensive procedure. Different parameters that go into the production of Zinculose were discussed in detail in a previous article [38]. Previously, researchers used pipetting [33,34,35] or placing paper in a suspension [37] to deposit zinc microparticles on paper. The main drawback of these methods is the lack of reproducibly depositing the same amount of zinc microparticles in the device as these particles rapidly settle down in any water suspension. Ferreira et al. [37] tried to overcome this drawback by weighing the disks being loaded with zinc before and after suspension; however, this is a very labor-intensive and time-consuming process as drying the disks takes 30 min. Another drawback is the availability of zinc microparticles to interact with the sample. The deposited zinc microparticles are left sitting on the surface of the material, which results in a non-ideal mixing with the sample that is flowing within the paper matrix. A third drawback is that these zinc microparticles are not bound to the paper and are free to flow into the detection zone, which can be detrimental to the performance of any 2D paper-based device. Use of Zinculose in paper-based devices overcomes these drawbacks.

### 2.3. Immobilized Griess Reagent 

Through a collaborative research effort between a chemistry and a mechanical engineering team funded by the National Science Foundation (NSF) to develop new paper-based devices for improving the detection of nutrients in water [45], the researchers developed multiple paper-based devices and tested them for the detection of nitrate using the Griess assay. However, the signal formed in the detection zone was non-uniform and showed a color gradient as the color was free to move with the flow of sample. Therefore, we decided that immobilizing the detection reagent would capture the color formed and would allow the flow of more sample over the detection zone, which should improve the performance of the device. This was recently successfully achieved by functionalizing one of the two reagents used in the Griess assay on paper, which resulted in an improvement in the detection limit of nitrite [39]. Functionalizing the detection zone is an equipment-free technique to concentrate the analyte of interest in a certain region by flowing a volume of sample that exceeds what is necessary to satisfy the hydrophilic zone [46]. This results in a very drastic improvement in the sensitivity of the device by improving the limit of detection and limit of quantification [47]. Incorporating a functionalized detection zone with an immobilized reagent to concentrate the analyte improved the detection limit of the device with the folding architecture by allowing the flow of a larger sample (Figure S35). 

### 2.4. Device Architecture 

**Figure 2:**
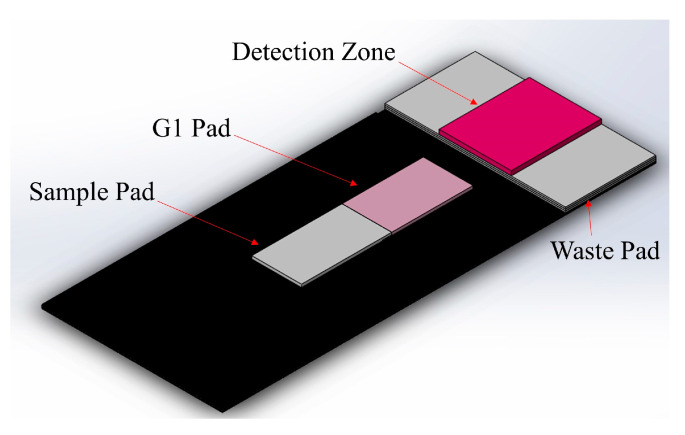
Device uses a simple mechanical folding architecture where the detection zone folds over the G1 pad. This provides the required delay step for nitrate to reduce to nitrite and allows for a uniform color formation in the detection zone. The G1 pad was either a strip of Zinculose for nitrate detection or chromatography paper (CHR1) for nitrite detection.

Several architectures were employed in the development of this device. Specifically, various fluidic valve strategies were initially explored in an attempt to control nitrate reduction time and improve its efficiency. Each of the different designs tested had its own set of advantages and disadvantages, which are mentioned in the Supplementary Information. Most of these architectures were used before the implementation of Zinculose and the functionalized detection zone. A lateral flow strip incorporating these two advancements was also developed (Figure S26); however, signal quality was an issue as there were streaking lines of color and a very distinctive color gradient over the detection zone. This was the case because of the color dispersion associated with horizontal flow [37]. Therefore, a folding device architecture was adopted (Figure 2). This allowed for a precise control mechanism for nitrate reduction and offered a very uniform color formation in the detection zone. This architecture was further optimized to improve the limits of detection and quantification of nitrate.

### 2.5. Device Preparation, Operation, and Analysis Procedure 

**Figure 3:**
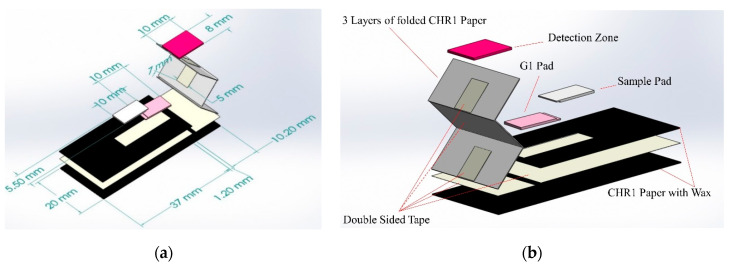
(**a**) Dimensions of the device and its components; (**b**) components of the paper-based microfluidic device.

The microfluidic device was designed using a vector graphics software (CorelDraw X6) and printed on chromatography paper (CHR1-GE Healthcare Whatman 1-3001878) using a solid ink wax printer (Xerox ColorQube 8570). This paper grade was used since it is made up of pure cotton cellulose fibers without any additives and is suitable for chemical applications. The device was then cut out using a laser engraver (Epilog mini 40W) and placed in an oven at 120 °C for 3 min to melt the printed wax and form hydrophobic surfaces. A guillotine cutter was used to cut sample pads from cellulose strips (Millipore CFSP203000). The waste pad was made up of 3 layers of chromatography paper, 10 × 20 mm in size. The different components of the microfluidic device were joined using a double-sided tape (0.0127 mm in thickness—FLEXmount 0.5 mil SELECT DF071736). A 5 × 7 mm double-sided tape was used in between the detection zone (8 × 10 mm) and the waste pad and also in-between the 3 layers of the waste pad to ensure continuity of fluid transfer. The components of the device and their dimensions are given in Figure 3. 

**Figure 4:**
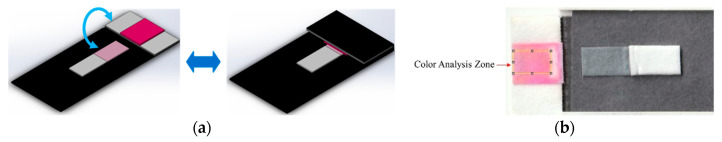
(**a**) The detection zone is folded over the G1 pad for 10 min. (**b**) Color analysis zone used in ImageJ to quantify the color intensity; this area is 105 by 175 pixels, which is approximately 4.5 by 7.5 mm.

Ninety-five microliter of sample is pipetted into the sample pad. The sample then flows to the G1 pad and nitrate molecules interact with the zinc microparticles and reduce to nitrite molecules. These nitrite molecules then react with the sulfanilamide to form diazonium ions. After an 11 min reduction time, the detection zone is folded over the G1 pad so that the diazonium ions can flow and react with the immobilized NED to form the colored azo dye (Figure 4a). A 1.25-inch paper binder clip is used to keep the device folded for 10 min to increase color formation and intensity in the detection zone. The binder clip is then removed, and the device is scanned using a desktop scanner (Canon TS6020) at a resolution of 600 DPI. Figure 4b shows the color analysis zone used in ImageJ, version 1.52a.

### 2.6. Reagents

The following reagents were used to collect the data for the results in this paper. Sulfanilamide (98%, Alfa Aesar-A1300136), citric acid (≥99%, Alfa Aesar-A103950B), sodium nitrate (≥99.5%, Honeywell Fluka-31440), sodium nitrite (≥99%, Honeywell Fluka-31443), and ASTM Type 1 deionized water (resistivity > 18 MΩ/cm, LabChem-LC267405). A real seawater sample from the Sargasso Sea region known for its low nutrient content [2,39,48] was used to see if the ions usually found in seawater have any effect on the performance of the device. This seawater sample was filtered through a 0.2 µm filter to remove any organic matter prior to its use. Zinculose strips were prepared using the procedure outlined in [38]. In short, cotton fibers from chromatography paper (CHR1-GE Healthcare Whatman 1-3001878) and zinc powder (99.3%, Fisher Chemical Zinc Certified Powder Z5-500) are mixed together in defined quantities and under controlled conditions to form a slurry that is then precipitated to produce a composite sheet. This sheet is then allowed to dry under room conditions and then cut into strips of required dimensions. The detection zones were cut from obtained cellulose strips with immobilized *N*-(1naphthyl)ethylenediamine (NED) [39]. All aqueous solutions were prepared using deionized water on the day of testing. The reagent solution used in the G1 pad was prepared with 50 mM sulfanilamide and 330 mM citric acid; these concentrations were successfully used in [33,49,50,51,52]. The device fabricated for detecting nitrate used a strip of Zinculose in the G1 pad whereas the device meant for detecting nitrite used a strip of chromatography paper instead. Chromatography paper or Zinculose strips were immersed in the reagent solution for 2 min to fully saturate before being allowed to air dry in room conditions for 2 h. The detection zone strips were immersed in a solution of 330 mM citric acid for 2 min before being allowed to air dry in room conditions for 2 h since this showed improved results (Figure S33). This result is in line with the requirements for the 2 reactions of the Griess assay, i.e., nitrite reacting with sulfanilamide and diazonium ions reacting with NED, to take place under acidic conditions [49]. Use of other acids such as hydrochloric, sulfuric, and phosphoric acids in the paper-based device was also investigated. However, hydrochloric acid completely evaporates without leaving any residue behind on paper so as to reproduce the required acidic conditions when the paper is rewet. This is in line with the results obtained by Cardoso et al. [53], who achieved a better detection result when the hydrochloric acid was added to the paper-based device after the addition of the sample. Sulfuric acid with the 0.5, 1, 2.2, 4.5, 6.6, and 8.8 M concentrations were tested. The 0.5 and 1 M concentrations made the chromatography paper very brittle and difficult to cut into size and fit into the paper-based device, whereas the higher concentrations of sulfuric acid tested did not dry on paper and so were impractical for use in the paper-based device. Citric acid gave better results than phosphoric acid and was therefore used.

### 2.7. Testing Range and Limits of Detection and Quantification

The concentrations tested were obtained by diluting a 1000 ppm solution freshly prepared on the day of testing by dissolving the required amount of nitrate or nitrite salt in deionized water. The following concentrations of 0, 0.01, 0.025, 0.05, 0.075, 0.1, 0.25, 0.5, 0.75, 1, 2.5, 5, 7.5, 10, 15, 25, and 50 ppm nitrate or nitrite were tested in deionized and seawater. Three samples per each concentration were tested in a completely randomized testing order. A MATLAB code was developed to fit the data to an exponential decay function of the form y = a × exp (−x/b) + c similar to the function used in [39] since we are using the detection zones they provided. The symbolic toolbox in MATLAB was used to calculate the limit of detection (LOD) and limit of quantification (LOQ) by finding the analyte concentration corresponding to the intensity values *y_LOD_* and *y_LOQ_* on the calibration curve using the following equations [54]:

(4) $$\mathrm{For}\mathrm{LOD}:y_{LOD}={\bar{y}}_{B}-3\sigma_{B}$$

(5) $$\mathrm{For}\mathrm{LOQ}:y_{LOQ}={\bar{y}}_{B}-10\sigma_{B}$$

where ${\bar{y}}_{B}$ is the mean color response of the blank and $\sigma_{B}$ is its respective standard deviation.

## 3. Results and Discussion

### 3.1. Signal Uniformity

**Figure 5:**
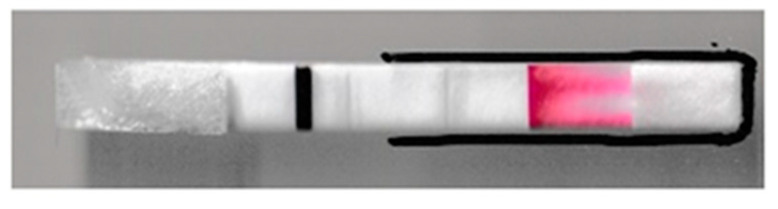
Lateral flow strip that was previously designed and tested. Color streaking and non-uniformity was observed in the detection zone.

The lateral flow strip architecture (Figure 5) that was previously developed in this work revealed that color first starts forming at random points of contact on the overlap between the G1 pad and the detection zone with the immobilized NED. We termed these sites as “seeding points” since color would preferentially continue to develop and become darker at these locations as more sample flowed through, with this being why color streaking and non-uniformity were observed in the detection zone (Figure S29). To overcome this drawback, we moved to a folding architecture design that allowed the detection zone to fold over the G1 pad, thus allowing the seeding points to be uniformly spread across a larger area on the detection zone, which resulted in a substantial enhancement in the uniformity of the signal obtained (Figure S30). Therefore, the area of the detection zone that directly overlaps the G1 pad was analyzed using ImageJ to quantify the color formed. Flood coating the detection zone with citric acid and allowing it to air dry improved the performance of the device (Figure S33). This is in agreement with the requirement of having the two reactions of the Griess assay take place under acidic conditions [49]. Moreover, as mentioned in Section 2.3, flowing more sample over the detection zone is an equipment-free method to enhance results. Therefore, we added a waste pad underneath the detection zone to permit the flow of more sample over it. This allowed concentrating the analyte in the detection zone and resulted in an improvement in the limit of detection and quantification and the production of reproducible results (relative standard deviation = 5.2%, *n* = 8).

### 3.2. Optimization of Device Parameters

To keep the number of experiments to run reasonable and manageable, we optimized the current platform using the traditional one-factor-at-a-time approach. First, the sample volume was optimized. Afterwards, the reduction time required to provide the darkest signal was then selected. Different zinc content in Zinculose were tested, and finally, the color development time to provide the darkest signal was chosen. Table 1 shows the testing range and optimum values of the different parameters investigated for the proposed paper-based device. The optimum value was selected as the testing condition that gave the lowest intensity value (darkest color formed) (Figures S36–S39). ImageJ assigns a value of 255 to the absolute white while it assigns a value of 0 to the absolute black. All other colors can be reproduced by a combination of the red, green, and blue components. The darker the color, the lower these values are. Oppositely, the lighter the color, the higher the values of the red, green, and blue components are. That is why the value of the sensor response (color intensity) decreases with increasing the concentration of analyte since the color becomes darker. The green component of the color shows the largest range of value with respect to change in the nitrate or nitrite concentration because the color formed is pinkish red in color. This means that the color mostly absorbed is the green component [37]. The red and blue components also show a difference in value, but the range of their change is not as large as that of the green. Previous researchers have also chosen the green intensity for their analysis of nitrate or nitrite [33,37,38,39]. Therefore, we also utilized green in our analysis. However, we normalized this green value by the summation of the red and blue components of the signal so as to capture the entire information of the color produced in the detection zone. 

### 3.3. Testing in Deionized Water

**Figure 6:**
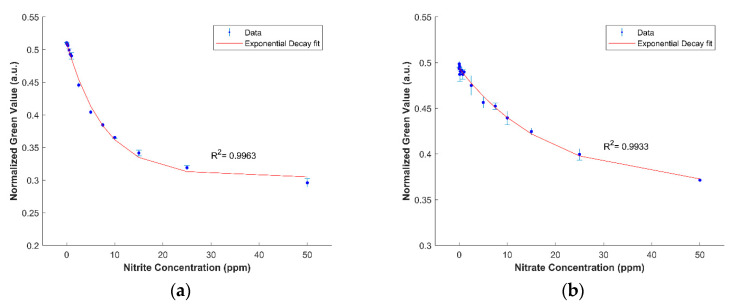
(**a**) An exponential decay calibration curve in the form y = a × exp (−x/b) + c, where a = 0.2059, b = 7.794, and c = 0.3047 was established for nitrite in deionized water. (**b**) An exponential decay calibration curve in the form y = a × exp (−x/b) + c, where a = 0.1299, b = 18.65, and c = 0.3638 was established for nitrate in deionized water. The error bars represent the standard deviation.

**Figure 7:**
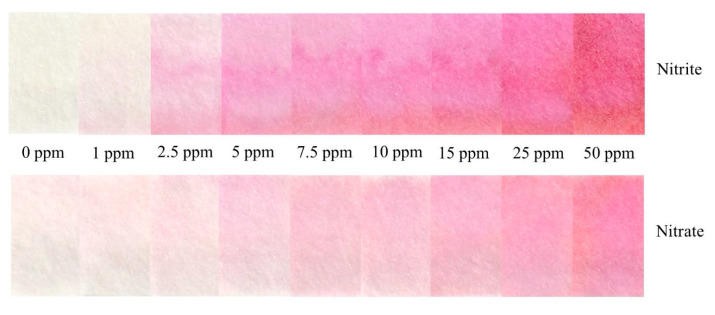
A color chart showing the evolution of color formation with increase in concentration.

Figure 6 shows the calibration curves developed for the detection of nitrate and nitrite in deionized water using the device. The limit of detection and quantification for nitrate are 0.533 ppm and 1.765 ppm, respectively, whereas the limit of detection and quantification for nitrite are 0.018 ppm and 0.061 ppm, respectively. A color chart showing the evolution of color formation as a function of concentration is provided for semi-quantitative analysis (Figure 7). There was a slight background signal in the nitrate detection for the 0 ppm condition. This was not observed in the detection zone of the nitrite device, even though they use the same immobilized reagents. Although the zinc microparticle assay used in making Zinuclose is of high purity (99.3%), there is also 0.001% “nitrogen compounds” in this zinc assay. Since nitrate is the most stable form of nitrogen in oxygenated environments [23,24], it is possible that this slight background signal comes from the nitrogen compounds accompanying the zinc microparticles. However, this signal was found to be very faint and did not significantly affect the results.

### 3.4. Testing in Sargasso Seawater

**Figure 8:**
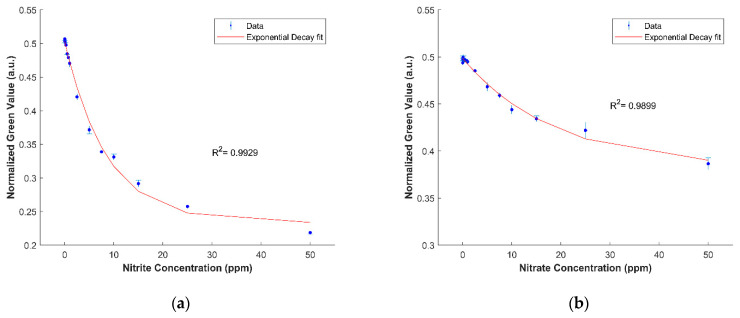
(**a**) An exponential decay calibration curve in the form y = a × exp (−x/b) + c, where a = 0.1164, b = 18.85, and c = 0.3819 was established for nitrite in Sargasso seawater. (**b**) An exponential decay calibration curve in the form y = a × exp (−x/b) + c, where a = 0.1164, b = 18.85, and c = 0.3819 was established for nitrate in Sargasso seawater. The error bars represent the standard deviation.

Figure 8 shows the calibration curves developed for the detection of nitrate and nitrite in Sargasso seawater using the device. The limit of detection and quantification for nitrate are 1.951 ppm and 5.135 ppm, respectively, whereas the limit of detection and quantification for nitrite are 0.025 ppm and 0.310 ppm, respectively.

### 3.5. Comparison of Results

Table 2 provides a comparison between the testing conditions and results achieved using the paper-based device developed in this work and those attained with paper-based devices fabricated previously. The limits of detection and quantification achieved were 55% and 41%, respectively, better than what has been previously achieved for the detection of nitrate in water using a paper-based device. This improvement can be attributed to the architecture utilized in addition to incorporating two new innovative materials in this device. The performance of the device developed in this work is also better than the other paper-based devices designed for the detection of nitrate in all other media except the ones designed by Ratnarathorn et al. [35] and Thongkam et al. [36]. Moreover, the results may be considered more accurate as the testing range encompasses the calculated limits of detection and quantification [39]. It is worth mentioning that while the limit of detection of the device developed in this work is higher than that achieved by the devices created in [35,36]—the range of application of the device developed in this work is larger, 0.01 to 50 ppm vs. 0.4 to 20 ppm and 0.5 to 40 ppm, respectively. Since the device developed in this study uses the same chemistry (zinc and Griess assay) as the devices previously developed for the detection of nitrate in food and saliva samples, we believe that this platform will also be applicable for these more complex matrices.

### 3.6. Device Portability, Longevity, and Commercialization

**Figure 9:**
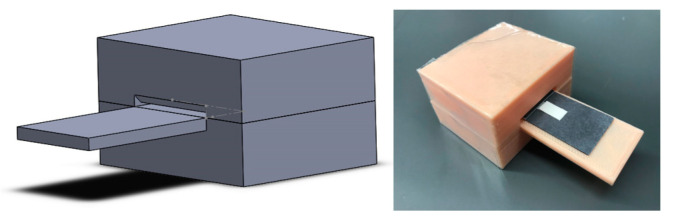
A 3D-printed holder that allows for a reproducible folding of the paper-based device. This holder can be integrated into a portable imaging platform that can be used in the field.

The developed paper-based device is very portable since it is only few centimeters in size. It is also very user-friendly and easy to use. The device can be easily incorporated into a portable imaging platform that allows the user to easily and reproducibly fold the device (Figure 9) and then analyze results in the field similar to what has been developed in [55,56,57]. These platforms are suitable for use in the field as they do not require any external power supply and they directly interface with a smartphone to provide instantaneous quantitative results. Although this study used a paper binder clip to keep the device folded for 10 min, the data show that there was no statistically significant difference between the results obtained by the binder clips and those obtained by the 3D holder (two-sample *t*-test at the 95% confidence level; *p* = 0.4 and DF = 6).

Device longevity depends on the stability of the different components making up the device. The zinc microparticles in Zinculose maintain their crystalline structure for over 6 months [38]. The detection zones with immobilized NED that have been flood coated with citric acid and stored in a desiccant box at room temperature, away from light and with a relative humidity less than 30% for 1 month, gave almost identical results to detection zones that have been freshly flood coated with citric acid on the day of testing (two-sample *t*-test at the 95% confidence level; *p* = 0.987 and DF = 4). Sulfanilamide oxidizes and changes color in a matter of days. This color degradation of sulfanilamide can be slowed down and the shelf life of the device improved by storing in nitrogen or under vacuum, away from light and under cold temperatures below 4 °C [33,35,37,39,58]. However, a chemistry approach to prohibit the oxidation and degradation of sulfanilamide needs to be further examined to aid the above engineering approaches. This would be similar to the case of commercial dip strips that utilize the Griess assay and have a shelf life of a couple of years when kept in their box under normal room conditions without necessitating sophisticated storage requirements.

These paper-based devices are relatively small in size and do not consume a large amount of material or use hazardous chemicals, making them environmentally friendly. The cost of each device is in the order of several U.S. cents only.

## 4. Conclusions

In this study, we followed an engineering approach to develop a highly sensitive paper-based device for the detection of nitrate in water. Several device architectures utilizing different valve alternatives were initially designed and tested. A simple paper-based design with a folding architecture was adopted. Folding the detection zone over the reagent pad improved the quality and uniformity of the signal developed in the detection zone as well as the detection limit. The device also incorporated two advancements in the field of paper-based technology—a new composite material improved the conversion efficiency of nitrate while the immobilized reagent allowed for more sample to flow through the detection zone. The limits of detection and quantification for the proposed nitrate device were 0.53 ppm and 1.18 ppm, respectively, in water. This represents 55% and 41%, improvement, respectively, than what has been previously achieved for the detection of nitrate in water using a paper-based device. Future work will include improving the shelf life of the device by enhancing the stability of the sulfanilamide by prohibiting its oxidation. Work will also include developing a suitable lightbox for use in the field. Additionally, analysis of nutrients in food samples is a very interesting area for further research.

## Figures and Tables

**Table 1:** Parameters investigated for the proposed paper-based device and their optimum values.

Parameter	Range Tested	Optimum Value
Sample volume (µL)	80–100	95
Reduction time (minutes)	10–15	11
Zinc content (mg/cm^2^)	0–30	20
Color development time (minutes)	1–15	10

**Table 2:** Comparison of results from this work with previous paper-based devices for detecting nitrate. * represents that nitrate and nitrite have the same results.

Reference	Nutrient	Media	Sample Volume (µL)	Testing Time (min)	Testing Range (ppm)	LOD (ppm)	LOQ (ppm)
This work	Nitrite	Water	95	21	0.01–50	0.018	0.061
	Nitrate					0.533	1.765
[33]	Nitrite	Water	20	4.5–8.5	0.46–6.9	0.046	0.359
	Nitrate				3.1–62	1.178	2.976
[34]	Nitrite	Food sample	80	12	2–10	1.2	4
	Nitrate				10–50	3.6	12
[35]	Nitrite *	Food sample	25	10	0.4–20	0.4	NA
	Nitrate						
[36]	Nitrite	Food sample	20	5	0.5–40	0.1	1.2
	Nitrate			10		0.4	1.4
[37]	Nitrite	Saliva	15	20–120	0.23–11.5	0.002	0.008
	Nitrate				12.4–74.4	4.96	16.74

All of the devices reported in this table use the Griess assay for detection. All of the devices reported used zinc for the reduction of nitrate, except [36], who used vanadium (III). Testing time is the total time required for reduction and signal analysis. NA, not available.

## Data Availability

Data is contained within the article or supplementary material. Additional data not presented in this article is available on request from the corresponding author.

## Supplementary Materials

The following are available online at https://www.mdpi.com/1424-8220/21/1/102/s1: Figure S1. Paper-based microfluidic device—R1. Figure S2. Paper-based microfluidic device architecture—R1. Figure S3. Components of R1. Figure S4. Dimensions of R1. Table S1. The parameters tested using paper-based microfluidic device. Table S2. Advantages and disadvantages of the R1 design. Figure S5. Paper-based microfluidic device—R2. Figure S6. Components of R2. Figure S7. Dimensions of R2. Figure S8. Fluidic testing of the wax valve. The device on the left has PBST before the wax valve and thus the valve “opens” after a certain reduction time and allows the fluid to flow into the detection zone. The device on the right does not have any PBST before the valve and thus the wax valve “holds” the fluid and does not allow it to pass into the detection zone. Figure S9. Comparison of R1 and R2. Figure S10. Average time it takes for a 0.25 mm wax valve to “open” and allow fluid to flow according to paper type. The paper types used in this test were (1) nitrocellulose HF09004XSS, (2) nitrocellulose HF09002XSS, and (3) Whatman filter paper grade 41. The error bars represent the standard deviation for four trials. Figure S11. Paper-based microfluidic device—R3. Figure S12. Design R3 uses a folding bridge to connect the different components of the device. It has three stacked reduction chambers. Figure S13. Dimensions of R3. Table S3. Advantages and disadvantages of the R3 design. Figure S14. Paper-based microfluidic device—R4. Figure S15. Design R4 is a simplified version of R3. It also uses a folding bridge to connect the different components of the device. However, it only uses one reduction chamber that can hold more zinc. Figure S16. Dimensions of R4. Figure S17. Fluidic testing of R4 with colored water. Color is not uniform in the detection zone. Figure S18. Paper-based microfluidic device—R5. Figure S19. Fluidic testing of R5 with colored water. Figure S20. Testing of R5 with a nitrate sample. Color was formed in the fluidic channel before the detection zone. There was a color gradient in the fluidic channel and no color was observed in the detection zone. Figure S21. Paper-based microfluidic device architecture—R5. Figure S22. Components of R5. Figure S23. R5 with a square sponge. The hydrophobic disk is filter paper that had wax printed using the solid ink wax printer and then melted to create a hydrophobic surface, whereas the hydrophilic disk is pure filter paper. Table S4. Advantages and disadvantages of the R5 design. Figure S24. Paper-based microfluidic device—R6. Figure S25. R6 design. Griess 1 is sulfanilamide and citric acid since the detection zone had immobilized NED. Figure S26. R6 with Zinculose that has two different zinc contents. Table S5. Advantages and disadvantages of the R6 design. Figure S27. A fabricated card that has all the components for the lateral flow strip. Strips are cut out of this card using a guillotine cutter. Figure S28. Paper-based microfluidic device—R7. Figure S29. Non-uniformity of color formed in the detection zone. We observed that color starts forming at random points along the overlap between the G1 pad and the detection zone with the immobilized NED. We termed these points as “seeding points” as color preferentially continues to develop and becomes darker at these locations as more sample flows through. That is why color streaking and non-uniformity is observed in the detection zone. Figure S30. The initial design with a folding detection zone architecture. The color produced in the detection zone is very uniform. Figure S31. Paper-based microfluidic device—R8. Figure S32. An exponential decay calibration curve in the form y = a. exp (-x/b) + c, where a = 0.184, b = 18.43, and c = 0.3162 was established for nitrite in deionized water. The error bars represent the standard deviation. The limit of detection and quantification were 0.227 ppm and 0.361 ppm, respectively. Figure S33. An exponential decay calibration curve in the form y = a. exp (-x/b) + c, where a = 0.202, b = 17.44, and c = 0.2981 was established for nitrite in deionized water. The error bars represent the standard deviation. The limit of detection and quantification were 0.191 ppm and 0.259 ppm, respectively. Figure S34. Paper-based microfluidic device—R9. Figure S35. An exponential decay calibration curve in the form y = a. exp (-x/b) + c, where a = 0.2059, b = 7.794, and c = 0.3047 was established for nitrite in deionized water. The error bars represent the standard deviation. The limit of detection and quantification were 0.018 ppm and 0.061 ppm, respectively. Figure S36. Signal vs. sample volume. The error bars represent the standard deviation for three trials. Figure S37. Signal vs. reduction time. The error bars represent the standard deviation for three trials. Figure S38. Signal vs. zinc content. The error bars represent the standard deviation for three trials. Figure S39. Signal vs. color development time. The error bars represent the standard deviation for three trials. Figure S40. Color formed in the detection zone vs. nitrate or nitrite concentration. References [46,47,49,59,60].
