# Supplementary material for: A New Paper-Based Microfluidic Device for Improved Detection of Nitrate in Water
Source: Sensors (Basel). 2020 Dec 26;21(1):102. doi: 10.3390/s21010102 (PMC7794956; doi:10.3390/s21010102)
Supplement: Supplementary file 1 [file sensors-21-00102-s001.pdf]

sensors

Article

A New Paper-Based Microfluidic Device for Improved Detection of Nitrate in Water

Amer Charbaji *, Hojat Heidari-Bafroui, Constantine Anagnostopoulos and Mohammad Faghri *

Microfluidics Laboratory, Department of Mechanical, Industrial and Systems Engineering, University of Rhode Island, 2 East Alumni Avenue, Kingston, RI 02881, USA; h_heidari@uri.edu (H.H.-B.); anagnostopoulos@uri.edu (C.A.)
* Correspondence: charbaji@uri.edu (A.C.); faghrim@uri.edu (M.F.)

Citation: Charbaji, A.; Heidari-Bafroui, H.; Anagnostopoulos, C.; Faghri, M. A New Paper-Based Microfluidic Device for Improved Detection of Nitrate in Water. *Sensors* **2020**, *21*, 102. https://dx.doi.org/10.3390/s21010102

Received: 5 November 2020
Accepted: 23 December 2020
Published: 26 December 2020

Publisher's Note: MDPI stays neutral with regard to jurisdictional claims in published maps and institutional affiliations.

Copyright: © 2020 by the authors. Licensee MDPI, Basel, Switzerland. This article is an open access article distributed under the terms and conditions of the Creative Commons Attribution (CC BY) license (http://creativecommons.org/licenses/by/4.0/).

Paper-based microfluidic device – R1:

The first paper-based microfluidic device (R1) designed had a wax valve printed on filter paper (Whatman grade 41). This wax valve acts as a delay valve to allow the nitrate sample to interact with the zinc microparticles in the reduction chamber and reduce to nitrite before it is allowed to flow through to the detection zone. Ahead of the wax valve there's a dried 2μL spot of PBST [1]. However, in [1], the material used for the platform was nitrocellulose (NC) and not filter paper. The valve actuation time was more reproducible and consistent in NC than in the case of filter paper, **Error! Reference source not found.** below. Nitrocellulose changed the color of the detection zone even in the case of a blank sample (0 ppm nitrite or nitrate) and therefore was not used in the device and replaced by filter paper.

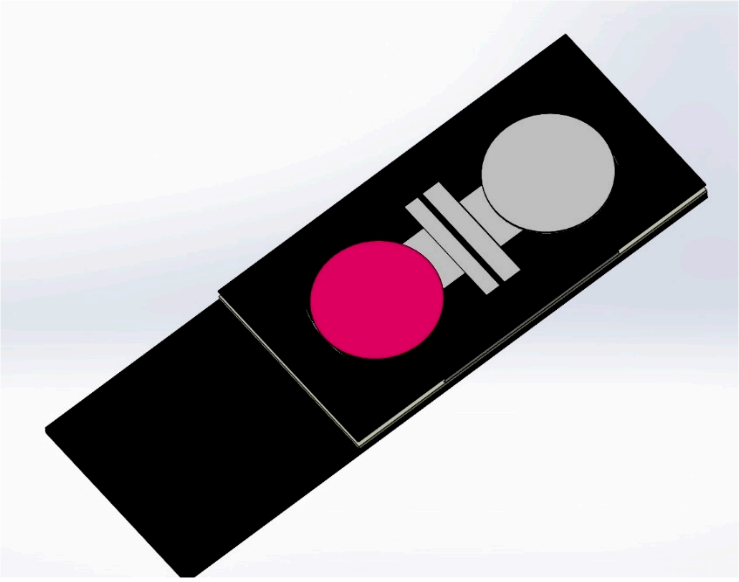

Figure S1. Paper-based microfluidic device - R1.

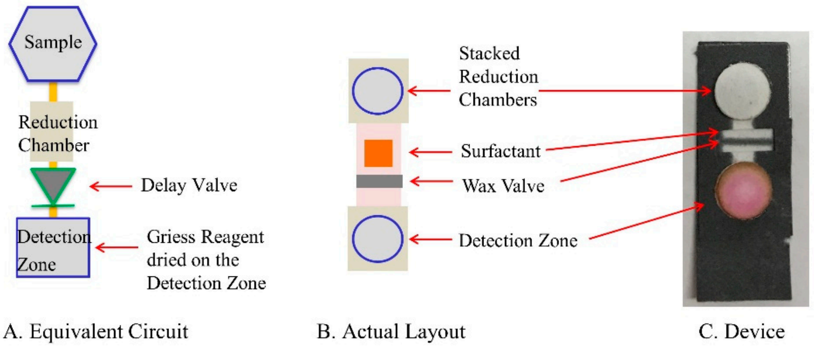

Figure S1. Paper-based microfluidic device architecture - R1.

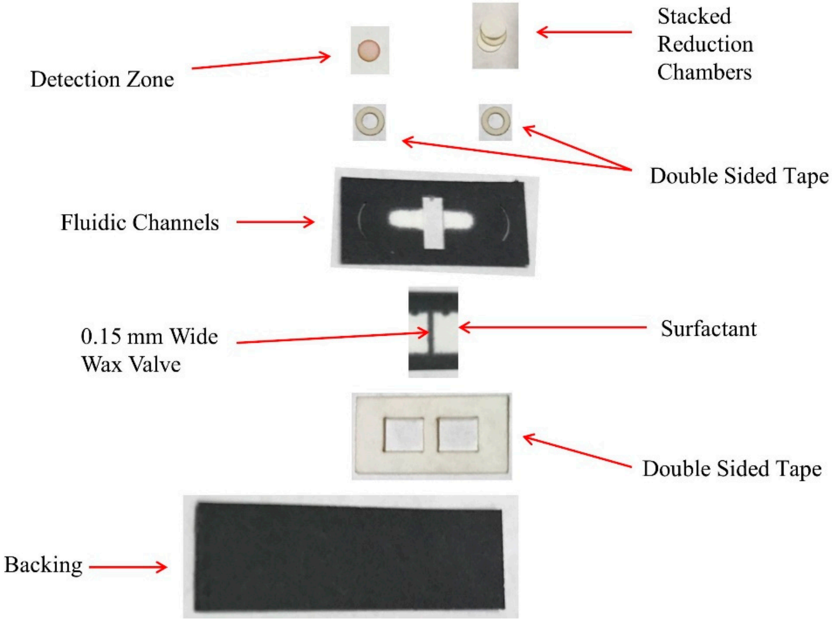

Figure S3. Components of R1.

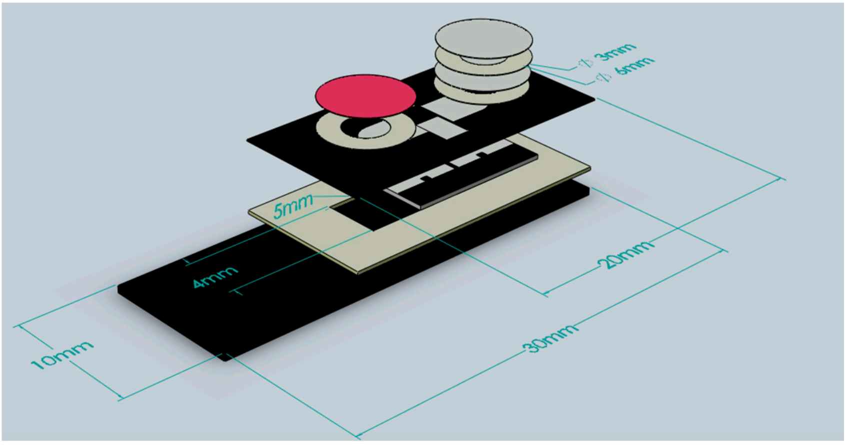

Figure S4. Dimensions of R1.

Table S1. The parameters tested using paper-based microfluidic device.

**Nitrate sample concentration (ppm)**	**100**
Sample volume (μL)	25
Mass of zinc deposited per reduction chamber (mg)	~ 2
Number of Reduction Chambers per device	3

Deposited volume of Griess Reagent (μL)	4
Griess Reagent drying time (min)	20
Scanning Time (min)	10–40

Table S2. Advantages and disadvantages of the R1 design.

Advantages	Disadvantages
Autonomous and easy to use: User has to only pipette the sample	The valve actuation time varies and is not exactly identical since it depends on the randomness in the paper. Humidity conditions played a major role on the repeatability of activation of the valve
Design is modular: The user can add as many reduction chambers as they please to improve conversion efficiency of nitrate	The wax valve is an actual barrier and makes the flow rate much slower when it is activated
-	The donut shaped double-sided tape in between the reduction chambers and the device is very tricky and takes a lot of time to peel
-	Pipetting the same amount of zinc in the reduction chamber is very difficult since zinc microparticles precipitate from the mixture
-	Color is not uniform in the detection zone. The fluid reaches the detection zone from the center and flows outward which creates a color gradient in the detection zone

Paper-based microfluidic device – R2:

The revised design of the paper-based microfluidic device (R2) also had a wax valve printed on filter paper. The design is very similar to R1. However, it is more simplified by having less components to prepare and thus making it easier and faster to fabricate. The set of advantages and disadvantages is almost identical to that of R1. The only difference is that the double sided tape is easier to peel and stick onto a larger area.

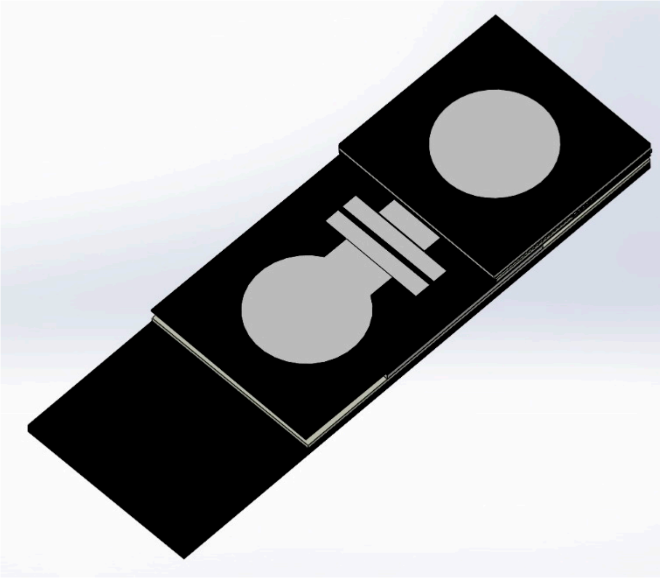

Figure S5. Paper-based microfluidic device – R2.

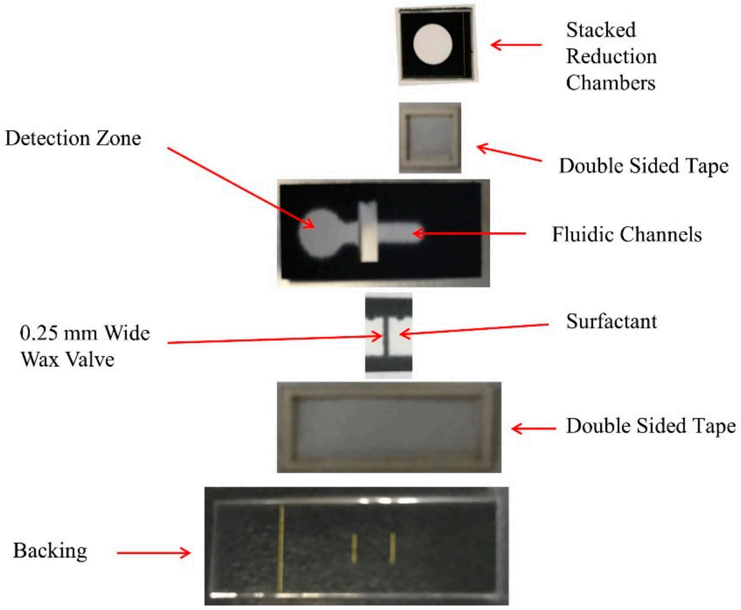

Figure S6. Components of R2.

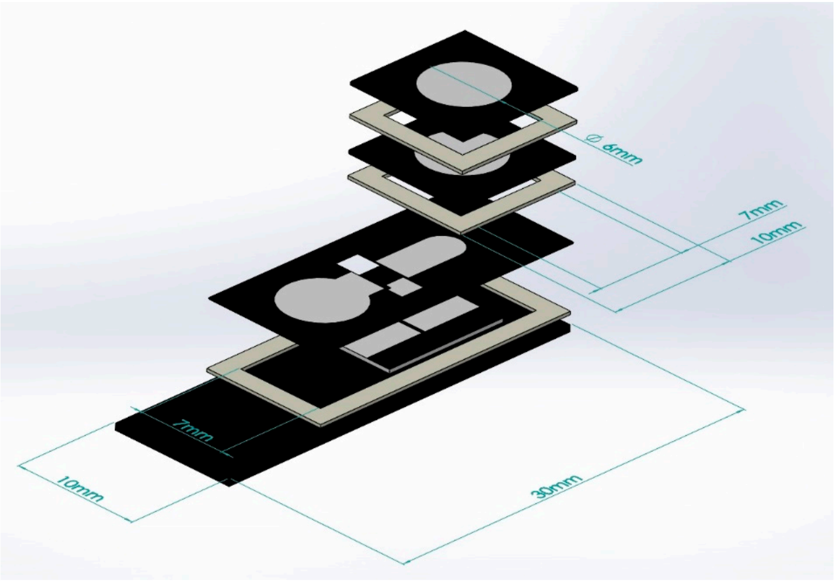

Figure S7. Dimensions of R2.

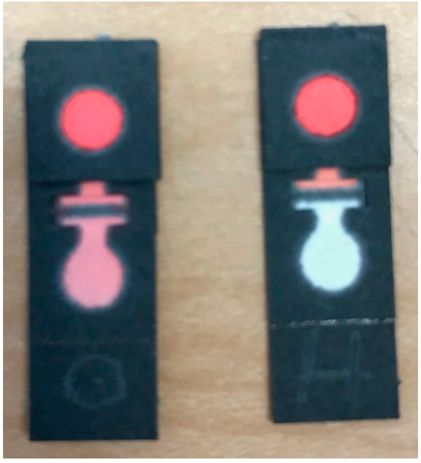

Figure S8. Fluidic testing of the wax valve. The device on the left has PBST before the wax valve and thus the valve "opens" after a certain reduction time and allows the fluid to flow into the detection zone. The device on the right does not have any PBST before the valve and so the wax valve "holds" the fluid and does not allow it to pass into the detection zone.

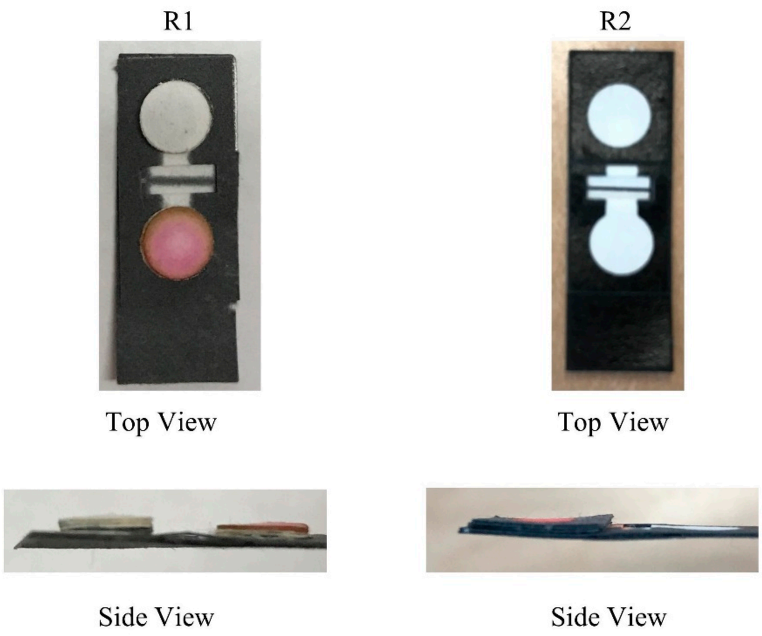

Figure S9. Comparison of R1 and R2.

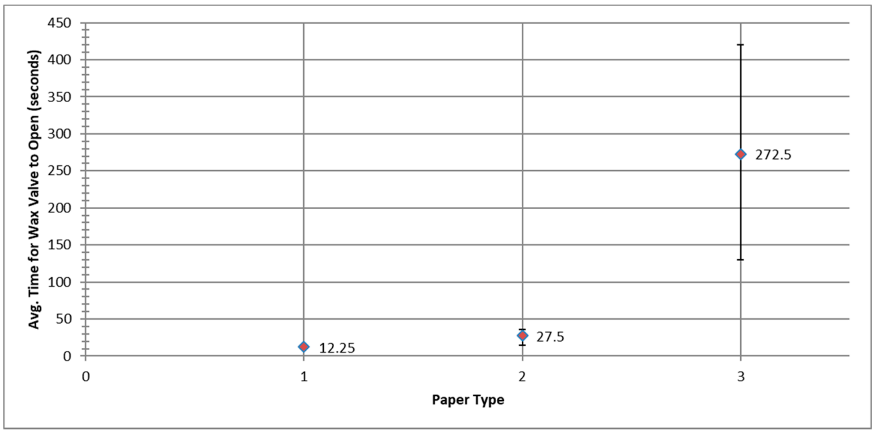

Figure S10. Average time it takes for a 0.25 mm wax valve to "open" and allow fluid to flow according to paper type. The paper types used in this test were (1) nitrocellulose HF09004XSS, (2) nitrocellulose HF09002XSS, and (3) Whatman filter paper grade 41. The error bars represent the standard deviation for four trials.

Paper-based microfluidic device – R3:

Since the wax valve was not giving reproducible results on filter paper and since we couldn't use nitrocellulose as part of the platform then we had to modify the design to include a mechanical valve which was a bridge that connected the reduction chamber to

the detection zone. The new microfluidic design (R3) included a folding bridge that would connect the different components of the device.

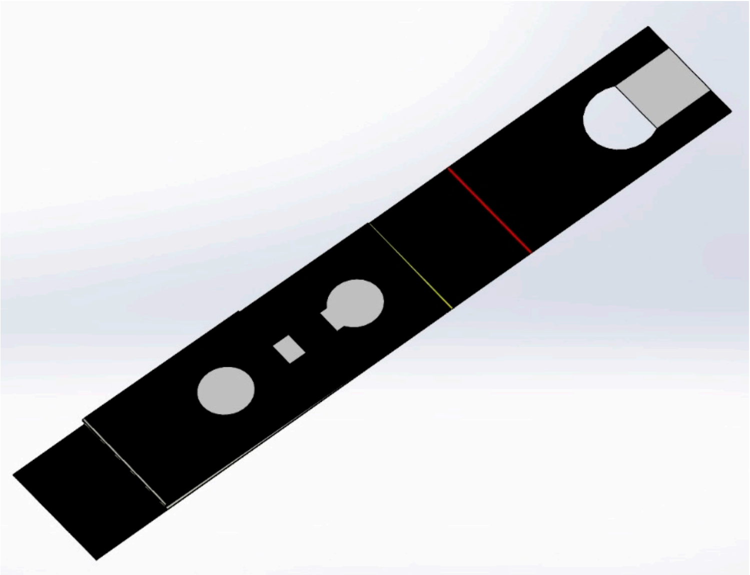

Figure S11. Paper-based microfluidic device – R3.

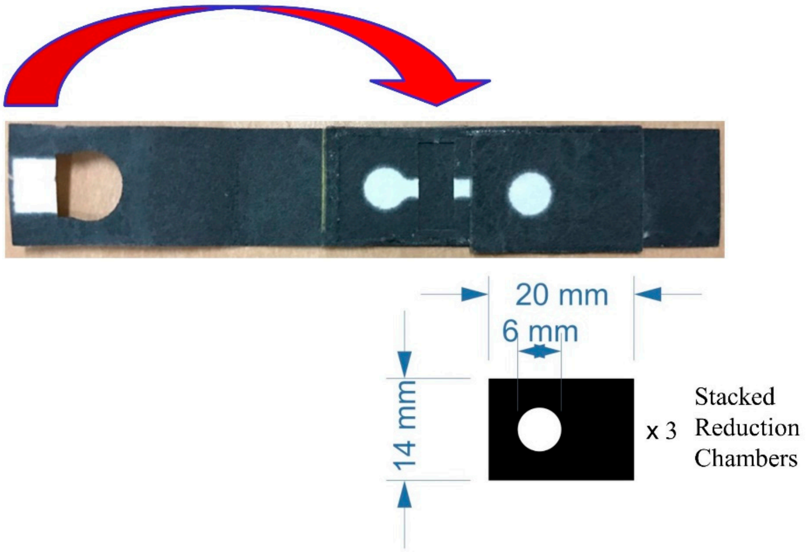

Figure S12. Design R3 uses a folding bridge to connect the different components of the device. It has three stacked reduction chambers.

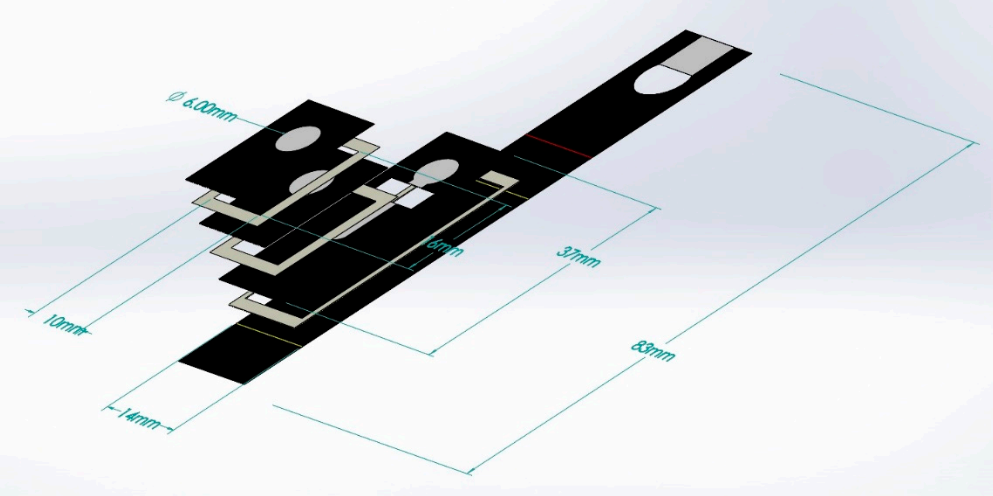

Figure S13. Dimensions of R3.

Table S3. Advantages and disadvantages of the R3 design.

Advantages	Disadvantages
Controlled actuation time. User will fold the bridge after a certain reduction time	It's not autonomous and requires the user to fold the bridge.
Design is easy to fabricate and easy to use	The bridge may not fold in the same location every single time which may affect the results
-	Pipetting the same amount of zinc in the reduction chamber is still a challenge
-	Color formed in the detection zone is not uniform

Paper-based microfluidic device – R4:

The R3 design was slightly modified to R4 to include 1 reduction chamber instead of 3. This made it easier to fabricate more devices in a shorter period of time. The connecting bridge was also made narrower so that it would be easier for the user to fold the bridge close to the connecting channel which would give more reproducible results. The set of advantages and disadvantages is identical to that of R3.

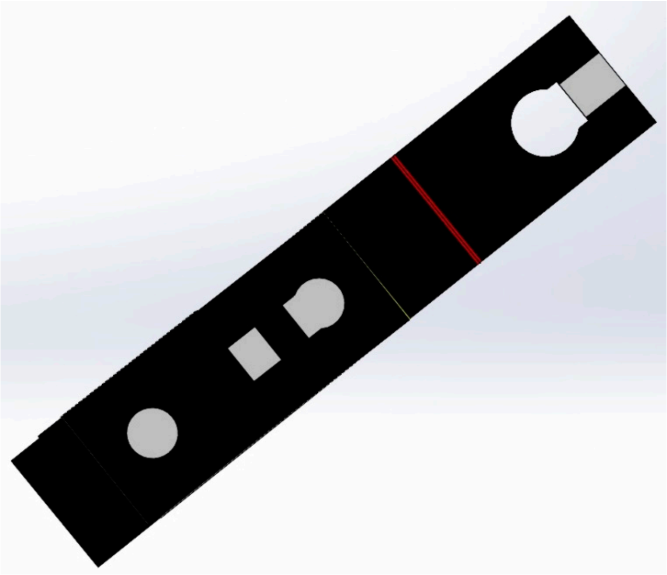

Figure S14. Paper-based microfluidic device – R4.

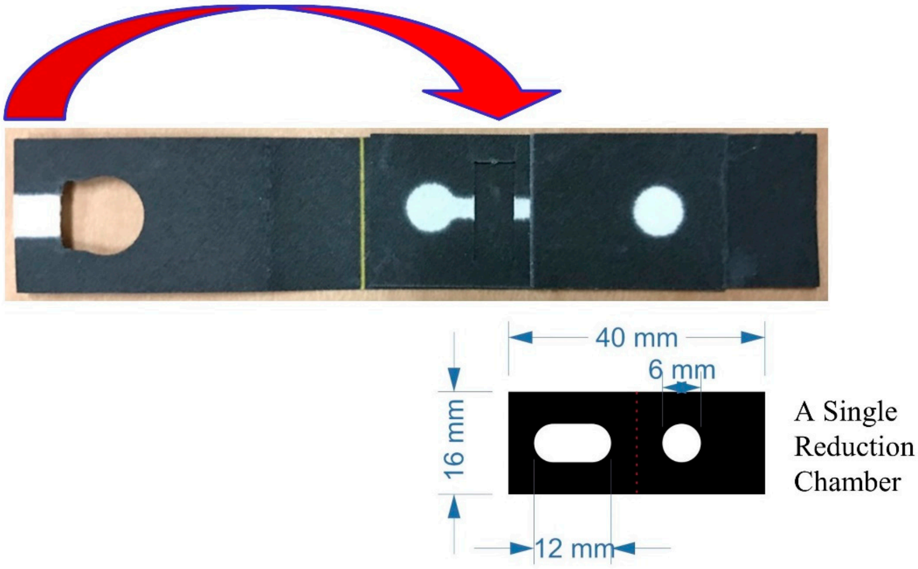

Figure S15. Design R4 is a simplified version of R3. It also uses a folding bridge to connect the different components of the device. However, it only uses one reduction chamber that can hold more zinc.

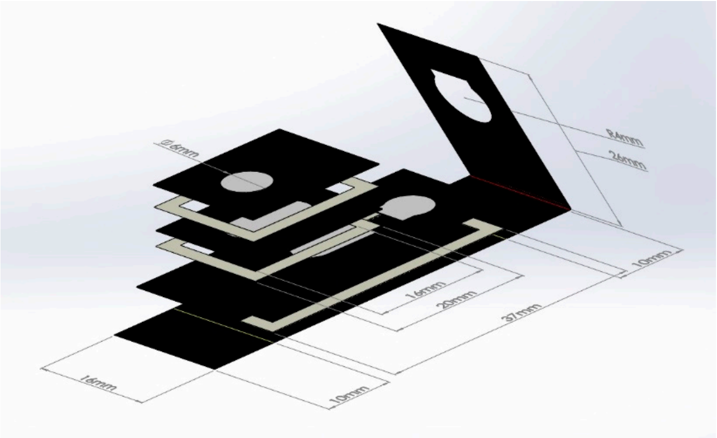

Figure S16. Dimensions of R4.

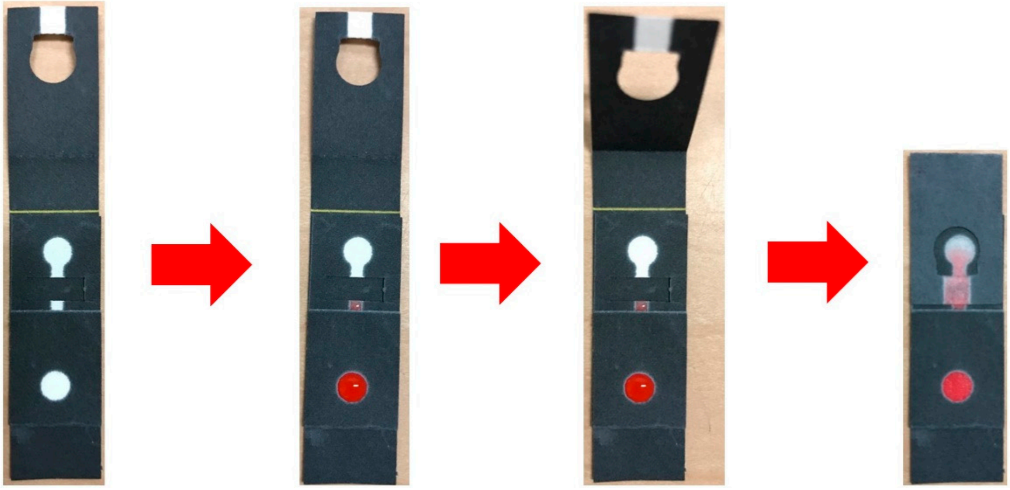

Figure S17. Fluidic testing of R4 with colored water. Color is not uniform in the detection zone.

Paper-based microfluidic device – R5:

The user error in folding the bridge prompted us to redesign the device to include a different valve mechanism. We wanted to keep the device simple and easy to use without requiring any external power source. So we decided to use a compressed sponge in design R5 to act as the valve actuation mechanism similar to [2] for autonomous operation of the device.

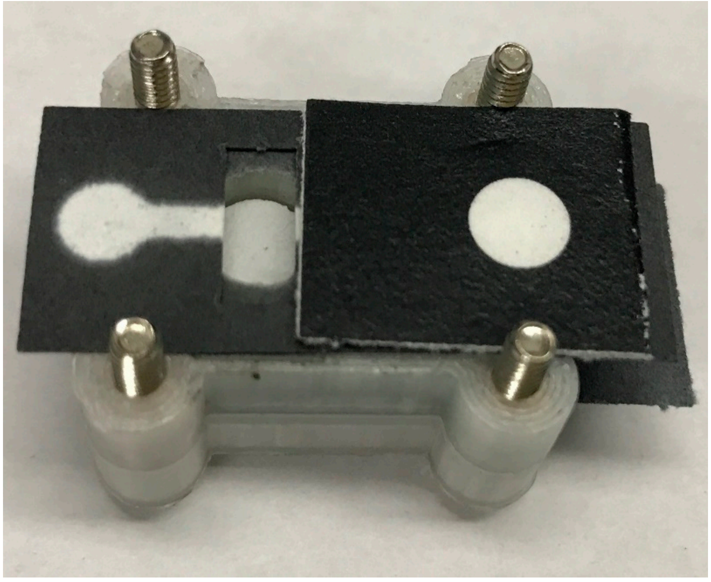

Figure S18. Paper-based microfluidic device – R5.

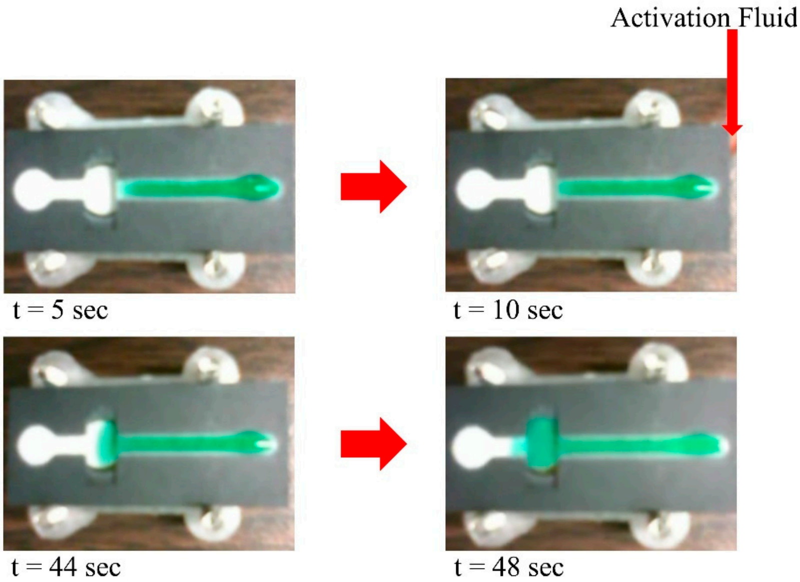

Figure S19. Fluidic testing of R5 with colored water.

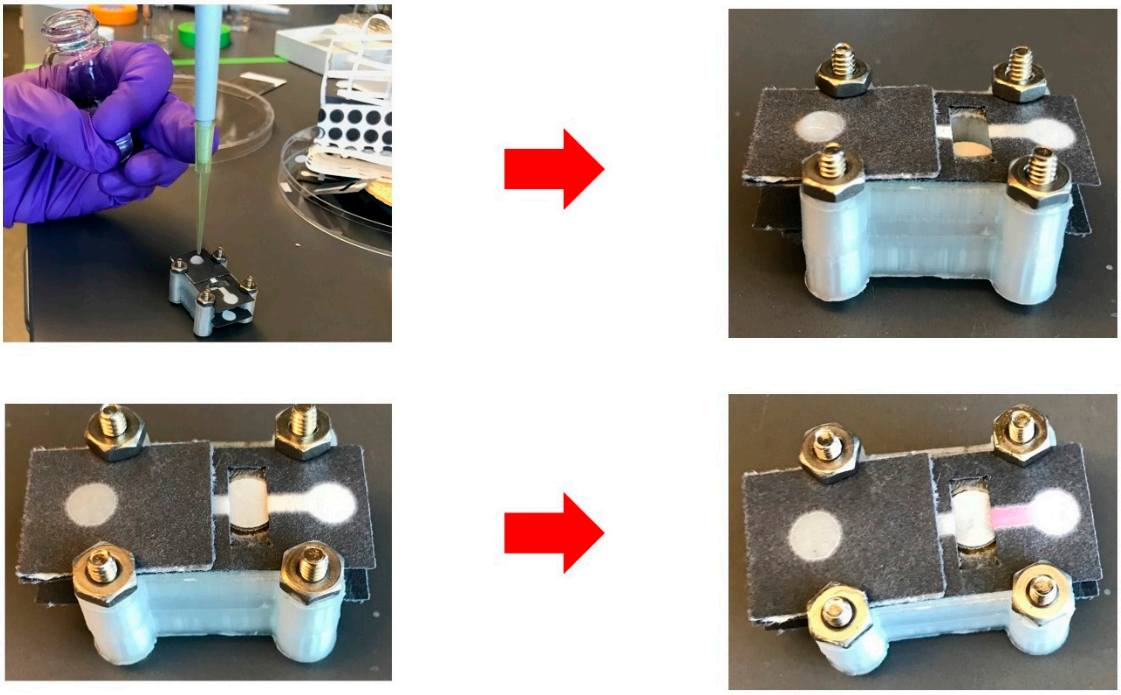

Figure S20. Testing of R5 with a nitrate sample. Color was formed in the fluidic channel before the detection zone. There was a color gradient in the fluidic channel and no color was observed in the detection zone. .

The sponge would sometimes get stuck in the shaft of the housing as it was expanding. This caused the device to have a non-repeatable activation time. The design was slightly modified to make the sponge square instead of being circular. However, this issue was still a problem that gave non-consistent activation times. The fluid transfer rate between the activation channel and the sponge played a very important role. We tried filter paper and glass fiber. We found that glass fiber was better than filter paper in transferring the fluid into the sponge for it to expand.

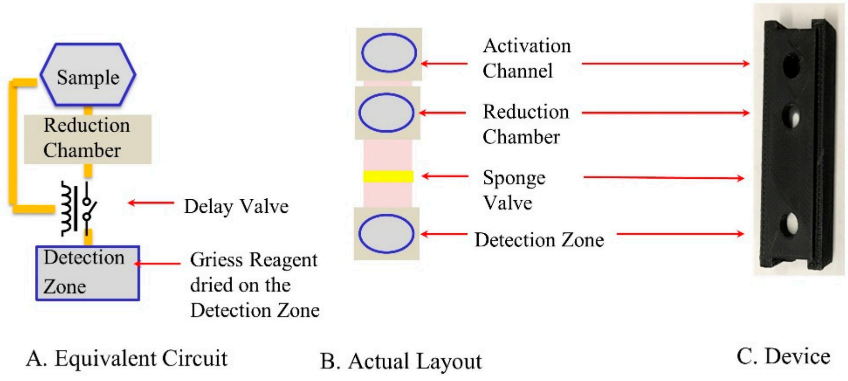

Figure S21. Paper-based microfluidic device architecture – R5.

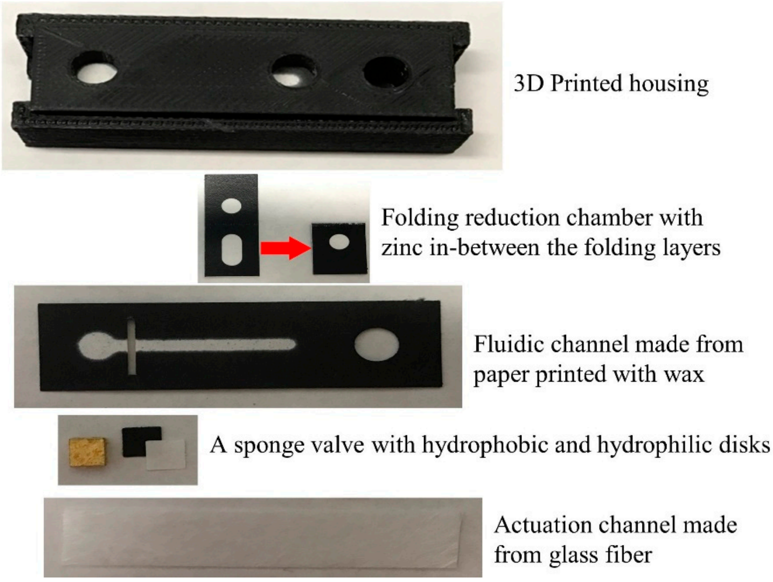

Figure S22. Components of R5.

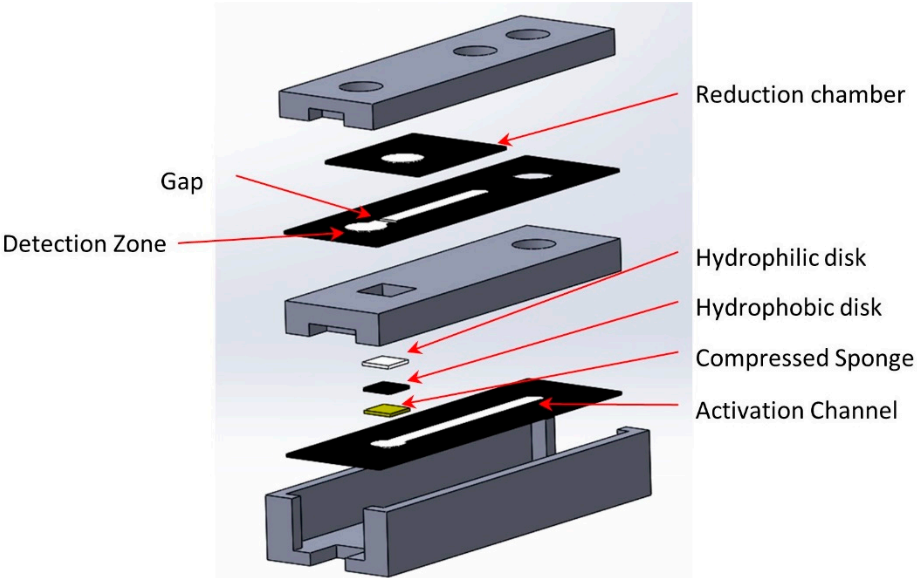

Figure S23. R5 with a square sponge. The hydrophobic disk is filter paper that had wax printed using the solid ink wax printer and then melted to create a hydrophobic surface. Whereas the hydrophilic disk is pure filter paper.

Table S4. Advantages and disadvantages of the R5 design.

**Advantages**	**Disadvantages**
Autonomous operation. User has to only pipette the sample at the start and the sponge valve will provide the required time delay for nitrate reduction.	The sponge didn't expand completely vertical on every run. It bent in some cases while it was expanding and got stuck in the shaft of the housing and therefore couldn't connect the channels. In other situations, it took longer to expand. So the activation time was difficult to control.

Design is easy to fabricate and easy to use.	The sponge required a large volume of fluid to expand. The fluid transfer rate between a paper activation channel and the sponge was too slow. The activation channel was replaced with glass fiber
-	Pipetting the same amount of zinc in the reduction chamber remains a challenge
-	Color formed in the fluidic channel before the detection zone and not in the detection zone itself

Paper-based microfluidic device – R6:

From the previous designs, it was clear that color formation was a critical issue since the color was not uniform and in a lot of cases formed in the fluidic connecting channel before the detection zone. Also, pipetting the same amount of zinc in the reduction chamber was very labor intensive and time consuming. Therefore, we implemented 2 new innovations in microfluidic paper-based technology in R6.

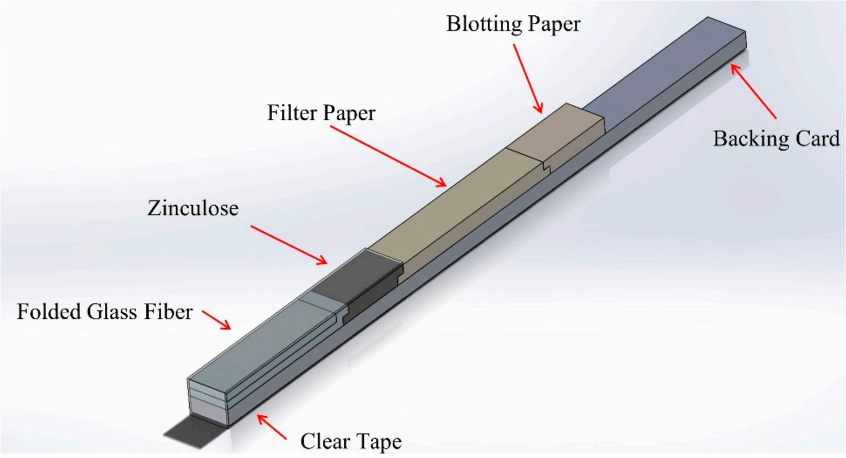

Figure S24. Paper-based microfluidic device – R6.

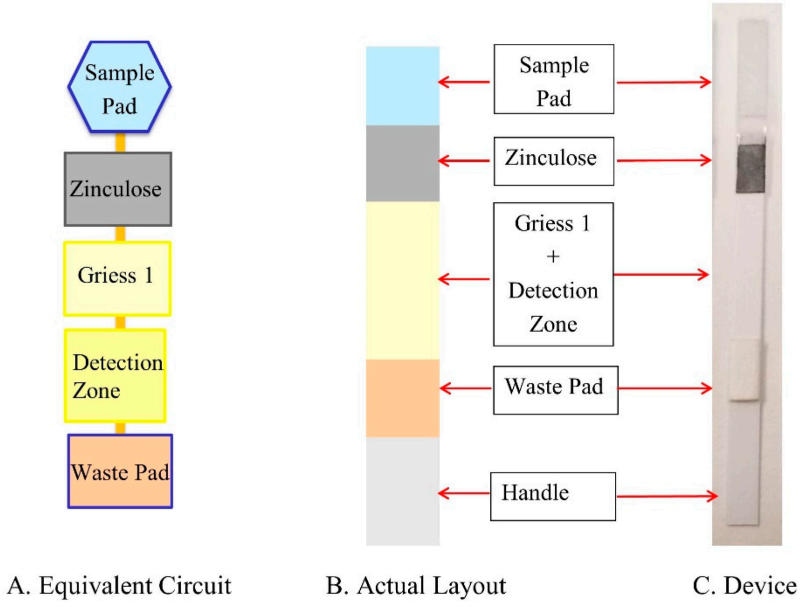

Figure S25. R6 design. Griess 1 is sulfanilamide and citric acid since the detection zone had immobilized NED.

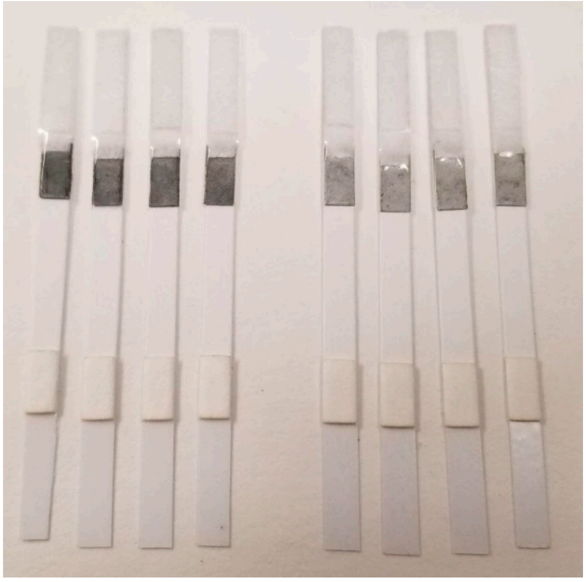

Figure S26. R6 with Zinculose that have two different zinc contents.

Table S5. Advantages and disadvantages of the R6 design.

Advantages	Disadvantages
Very easy to fabricate. Fabrication is done by preparing a card that has all the different components, **Error! Reference source not found.**. The card is then cut using a guillotine cutter into several lateral flow strips	The color formed in the detection zone was not uniform

Very easy to use. The user just needs to dip the sample pad in the solution for few seconds	The lateral flow strip was too long
Zinculose provided the reduction capability to reduce nitrate to nitrite. There was no longer a need to prepare the reduction chamber for each device individually	-

Paper-based microfluidic device – R7:

R7 is almost identical to the architecture of R6. However, it was shorter and there were separate regions for G1 (sulfanilamide and citric acid) and the detection zone that was functionalized with immobilized NED. This increased the longevity of the device as placing G1 solution on the immobilized NED turned the color of the paper slightly pinkish as it dried. The advantages and disadvantages for R7 are the same as those stated for R6.

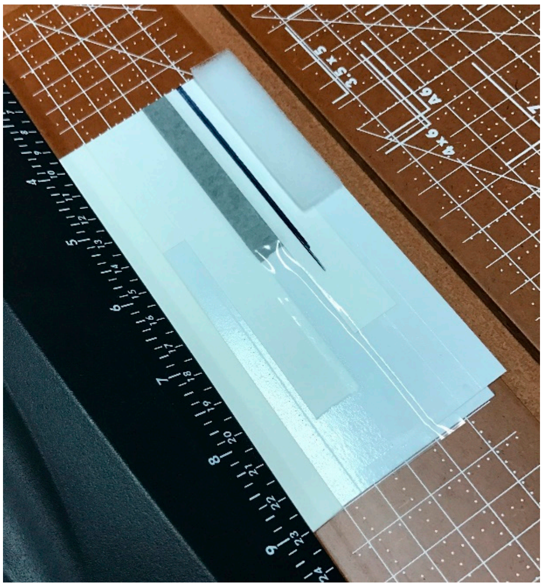

Figure S27. A fabricated card that has all the components for the lateral flow strip. Strips are cut out of this card using a guillotine cutter.

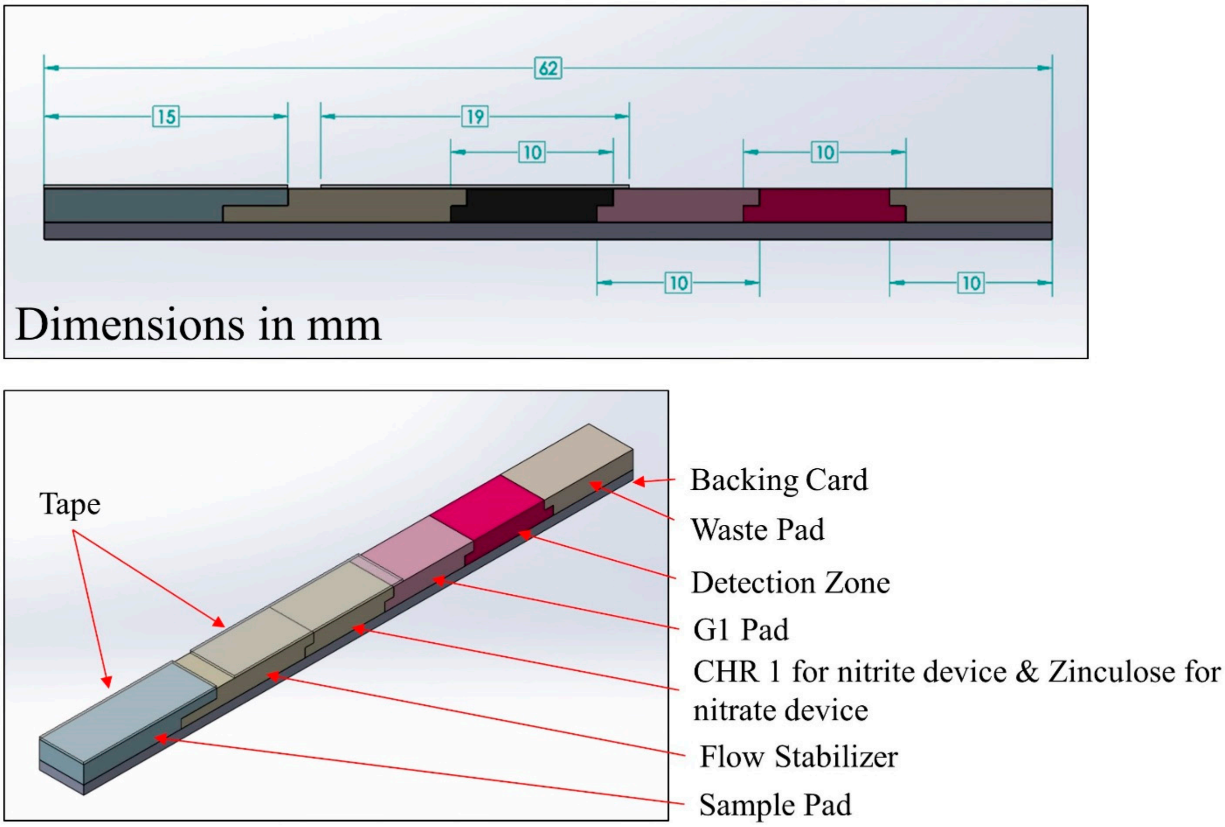

Figure S28. Paper-based microfluidic device – R7.

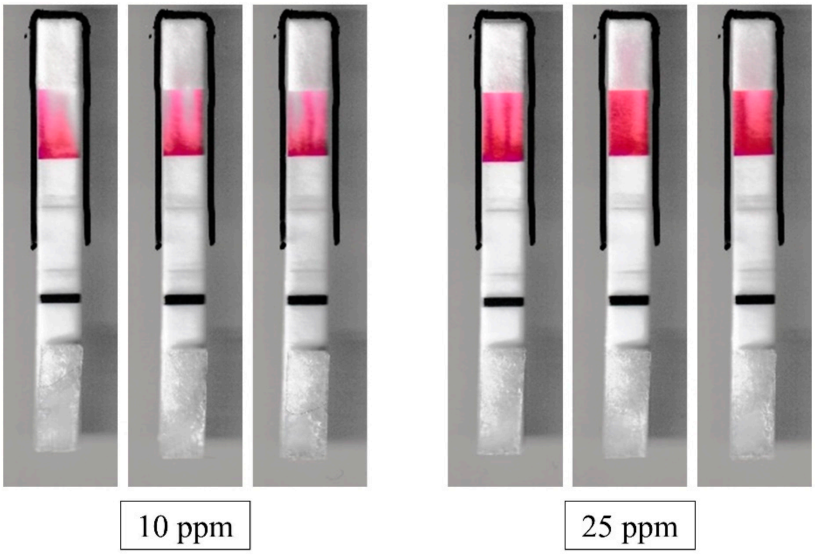

Figure S29. Non-uniformity of color formed in the detection zone. We observed that color starts forming at random points along the overlap between the G1 pad and the detection zone with the immobilized NED. We termed these points as "seeding points" as color preferentially continues to develop and becomes darker at these locations as more sample flows through. That is why color streaking and non-uniformity is observed in the detection zone.

Paper-based microfluidic device – R8:

Due to the non-uniformity in the color formed in the later flow architecture and the color gradient that forms in the detection zone, we decided to use a folding device design to produce a more uniform color in the detection zone.

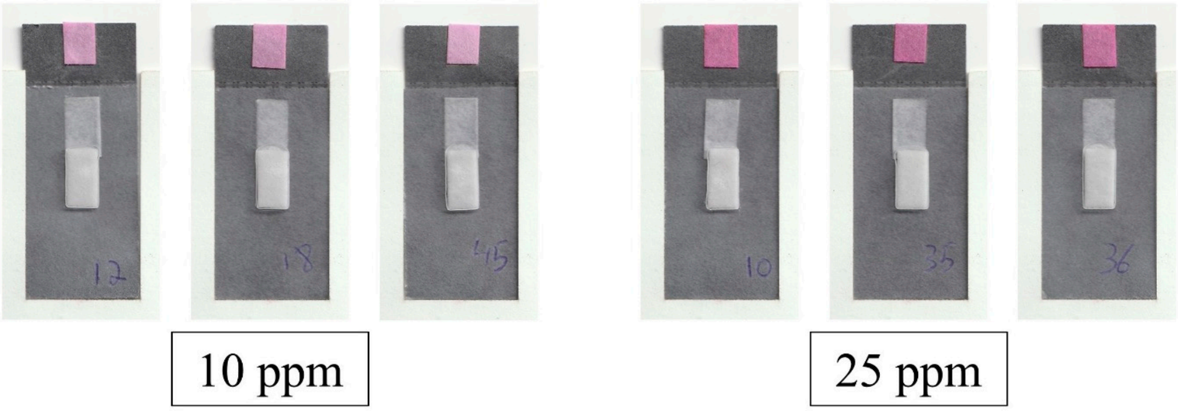

Figure S30. The initial design with a folding detection zone architecture. The color produced in the detection zone is very uniform.

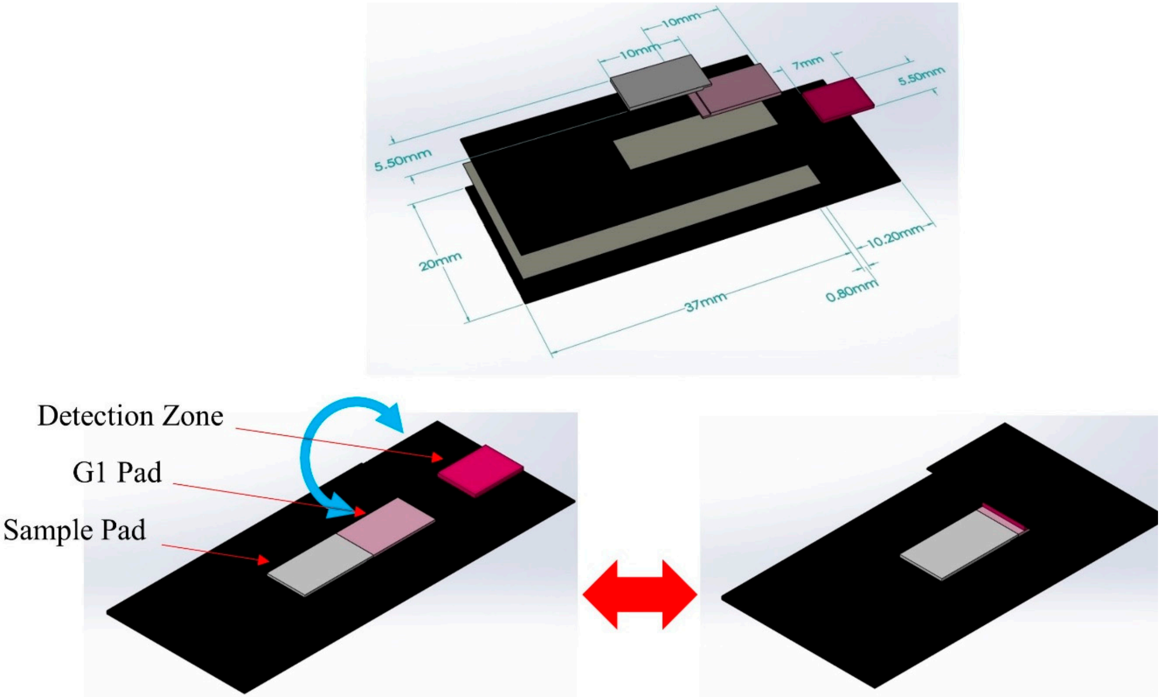

Figure S31. Paper-based microfluidic device – R8.

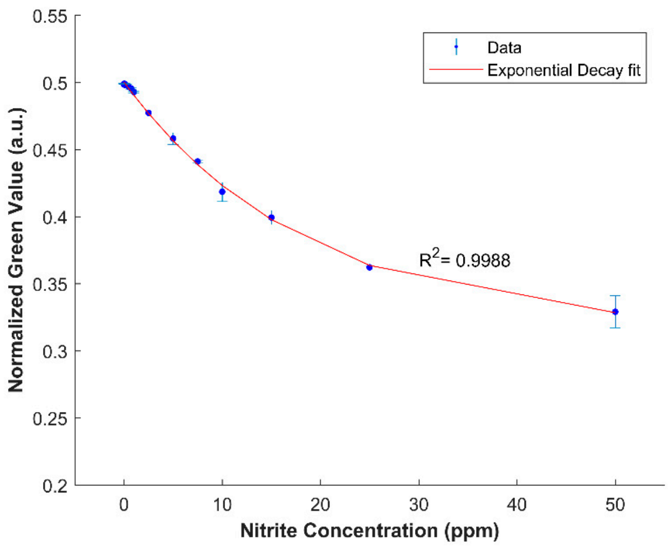

Figure S32. An exponential decay calibration curve in the form y = a × exp (-x/b) + c where a = 0.184, b = 18.43 and c = 0.3162 was established for nitrite in deionized water. The error bars represent the standard deviation. The limit of detection and quantification were 0.227 ppm and 0.361 ppm respectively.

The Griess assay involves two reactions that take place under acidic conditions [3]. So we flood coated the detection zones that were functionalized (immobilized NED) with citric acid 330 mM for 2 minutes and allowed to air dry before using in the device. This improved the performance of the device by lowering the limits of detection and quantification.

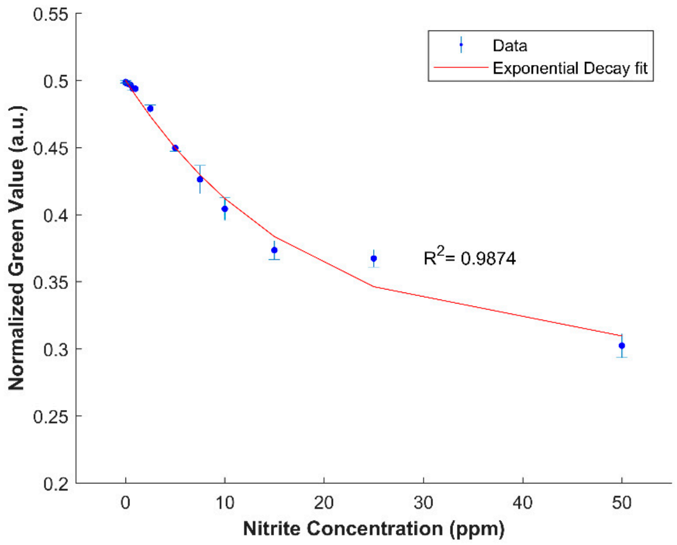

Figure S33. An exponential decay calibration curve in the form y = a . exp (-x/b) + c where a = 0.202, b = 17.44 and c = 0.2981 was established for nitrite in deionized water. The error bars represent the standard deviation. The limit of detection and quantification were 0.191 ppm and 0.259 ppm respectively.

Paper-based microfluidic device – R9:

The signal formed in the detection zone of R8 is very uniform and there's no color gradient similar to what was observed in the detection zone of R7. However, R7 allows the passage of a larger volume of sample of the detection zone. Passing a larger volume of sample over a detection zone with immobilized reagent is known to concentrate the

analyte of interest [4] and improve the limits of detection and quantification [5]. Therefore, R8 was modified to include a waste pad that would allow the passage of a larger volume of sample over the detection zone.

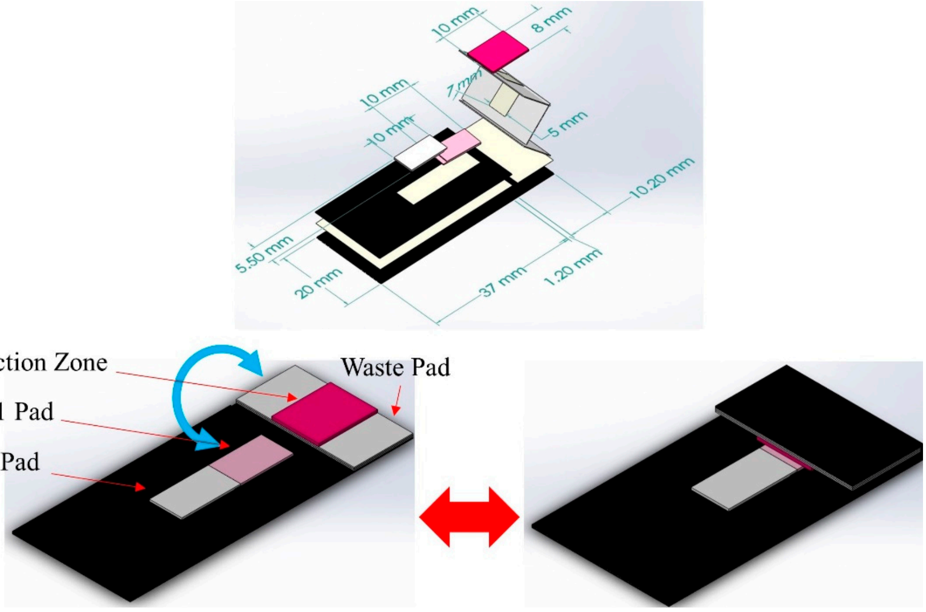

Figure S34. Paper-based microfluidic device – R9.

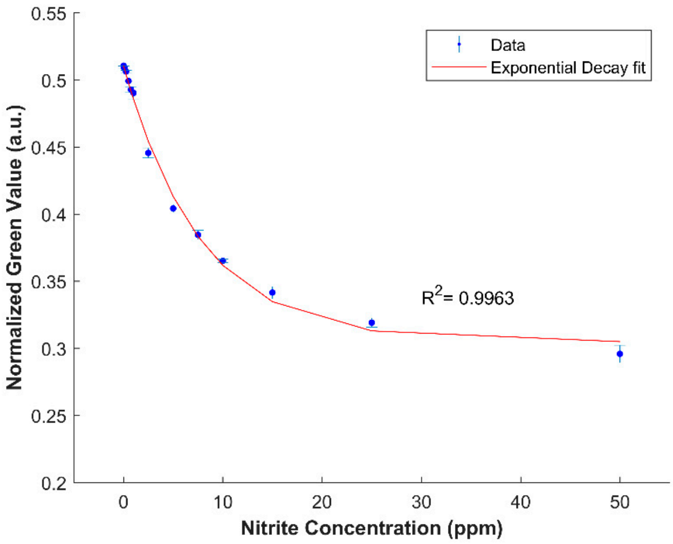

Figure S35. An exponential decay calibration curve in the form y = a × exp (-x/b) + c where a = 0.2059, b = 7.794 and c = 0.3047 was established for nitrite in deionized water. The error bars represent the standard deviation. The limit of detection and quantification were 0.018 ppm and 0.061 ppm respectively.

Optimization of the different parameters for device operation:

Sample Volume:

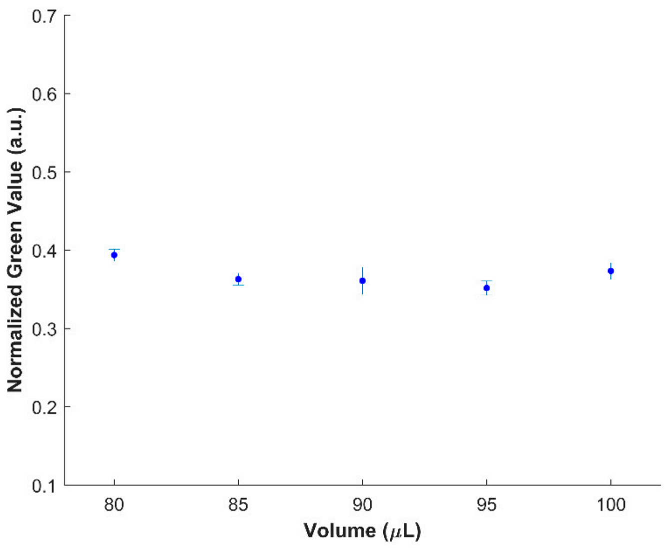

Figure S36. Signal vs. sample volume. The error bars represent the standard deviation for three trials.

Reduction Time:

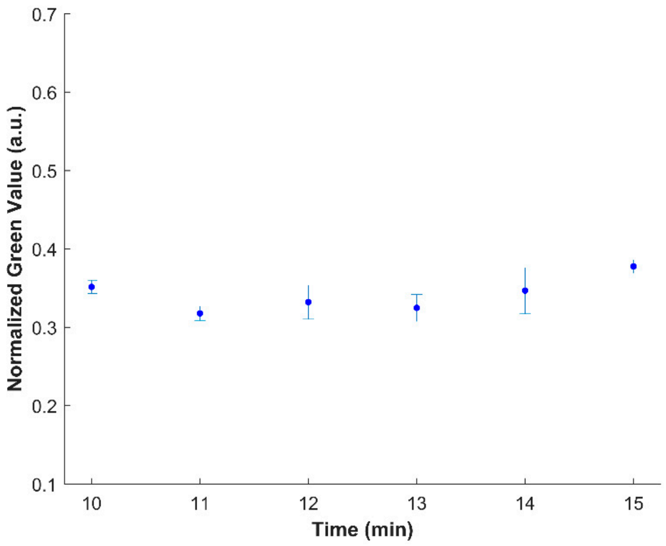

Figure S37. Signal vs. reduction time. The error bars represent the standard deviation for three trials.

Zinc Content:

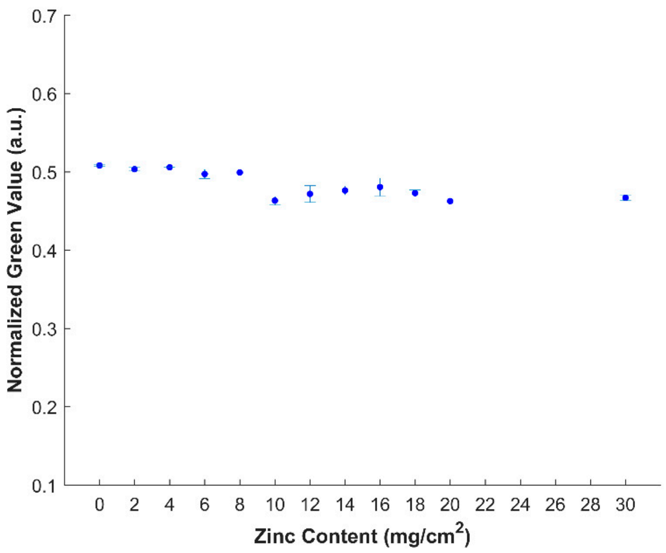

Figure S38. Signal vs. zinc content. The error bars represent the standard deviation for three trials.

Color Development Time:

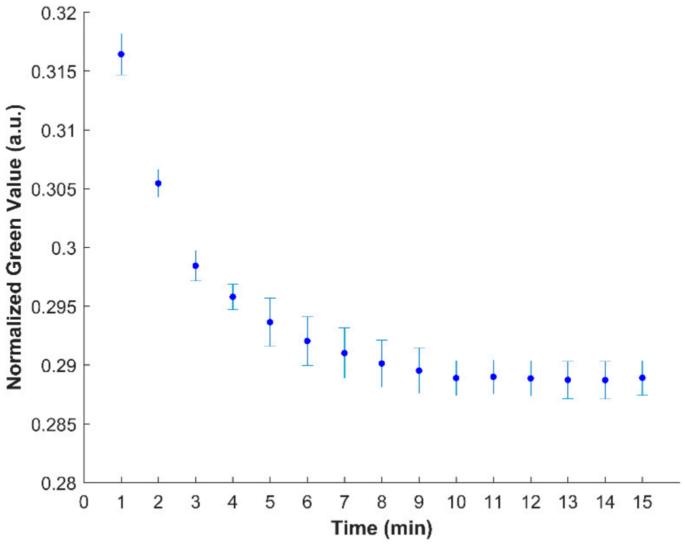

Figure S39. Signal vs. color development time. The error bars represent the standard deviation for three trials.

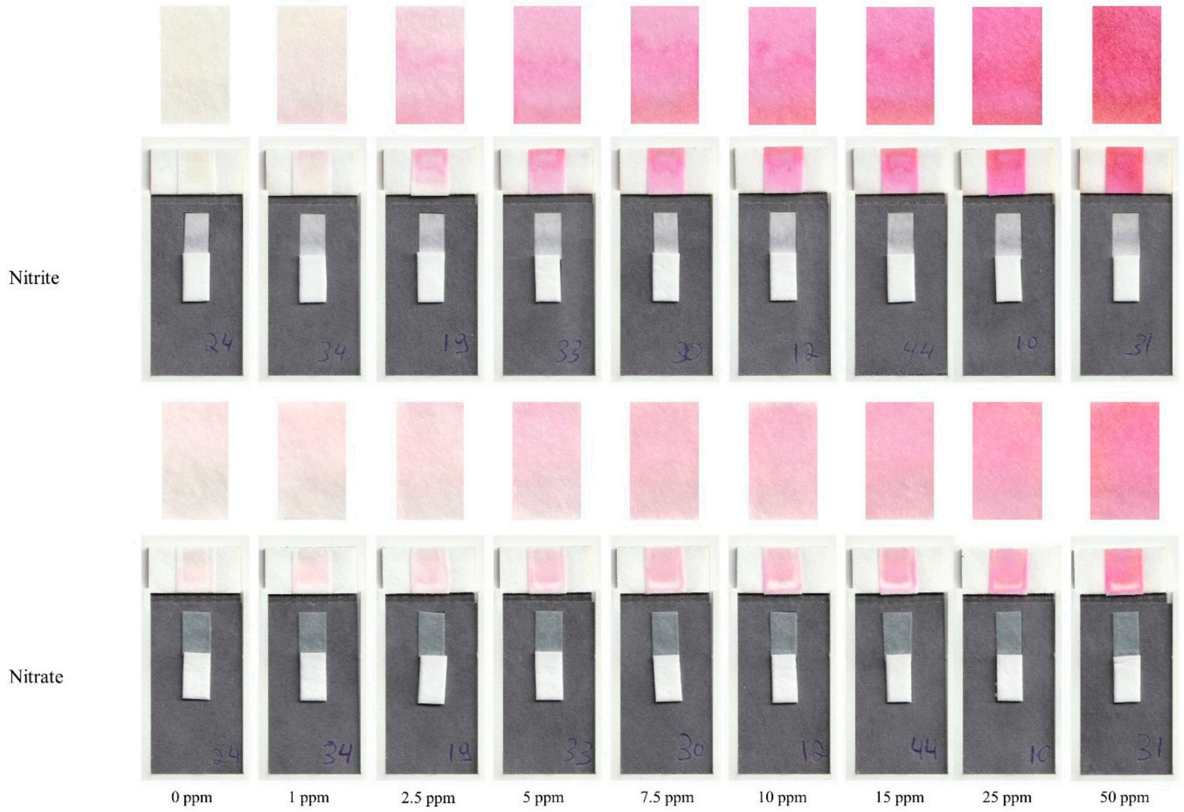

Figure S40. Color formed in the detection zone vs. nitrate or nitrite concentration.

References

(1) Lai, Y.T.; Tsai, C.H.; Hsu, J.C.; Lu, Y.W. Microfluidic Time-Delay Valve Mechanism on Paper-Based Devices for Automated Competitive ELISA. *Micromachines* **2019**, *10*, 837, doi: 10.3390/mi10120837.

(2) Toley, B.J.; Wang, J.A.; Gupta, M.; Buser, J.R.; Lafleur, L.K.; Lutz, B.R.; Fu, E.; Yager, P.A. Versatile Valving Toolkit for Automating Fluidic Operations in Paper Microfluidic Devices. *Lab Chip* **2015**, *15*, 1432–1444, doi:10.1039/c4lc01155d.

(3) Weng, C.H.; Chen, M.Y.; Shen, C.H.; Yang, R.J. Colored Wax-Printed Timers for Two-Dimensional and Three-Dimensional Assays on Paper-Based Devices. *Biomicrofluidics* **2014**, *8*, 066502, doi:10.1063/1.4902246.

(4) Kudo, H.; Yamada, K.; Watanabe, D.; Suzuki, K.; Citterio, D. Paper-Based Analytical Device for Zinc Ion Quantification in Water Samples with Power-Free Analyte Concentration. *Micromachines* **2017**, *8*, 127, doi:10.3390/mi8040127.

(5) Shimada, Y.; Kaneta, T. Highly Sensitive Paper-Based Analytical Devices with the Introduction of a Large-Volume Sample via Continuous Flow. *Anal. Sci.* **2018**, *34*, 65–70, doi:10.2116/analsci.34.65.
